# Metabolomic Signatures and Predictive Utility of *LOXL1*-Associated Genetic Risk Scores for Exfoliation Syndrome/Glaucoma in US Cohorts

**DOI:** 10.3390/metabo15090582

**Published:** 2025-08-30

**Authors:** Namuunaa Juramt, Oana A. Zeleznik, Louis R. Pasquale, Janey L. Wiggs, Jae H. Kang

**Affiliations:** 1 Channing Division of Network Medicine, Department of Medicine, Brigham and Women’s Hospital and Harvard Medical School, Boston, MA 02115, USA; nhotz@channing.harvard.edu (O.A.Z.); nhjhk@channing.harvard.edu (J.H.K.); 2 Department of Ophthalmology, Icahn School of Medicine at Mount Sinai, New York, NY 10029, USA; louis.pasquale@mssm.edu; 3 Department of Ophthalmology, Harvard Medical School, Massachusetts Eye and Ear Infirmary, Boston, MA 02115, USA; janey_wiggs@meei.harvard.edu

**Keywords:** metabolomics, genetic risk score, exfoliation syndrome, glaucoma, *LOXL1*

## Abstract

Background: Exfoliation syndrome (XFS) is a form of deleterious ocular aging mediated by genetic and environmental factors that frequently produces glaucoma (XFG). We aimed to develop a genetic risk score (GRS), assess its clinical utility, and identify metabolites/metabolite classes associated with a high GRS. Methods: For 39,472 Nurses’ Health Studies (NHS, 1980–2018; NHS2, 1989–2019) and Health Professionals Follow-up Study (1986–2018) participants aged ≥ 40 years reporting eye exams and no baseline glaucoma, we formed an eight-single nucleotide polymorphism Genetic Risk Score (GRS8) using loci with genome-wide associations with XFS. We estimated relative risks (RR) for incident XFG suspect (XFGS)/XFG (n = 118 cases) and Harrell’s C statistics. Among 7547 participants with plasma metabolites measured via liquid chromatography-mass spectrometry, we evaluated the relation between GRS8 and 427 individual metabolites and 20 metabolite classes, adjusting for multiple comparisons. Results: Higher GRS8 was associated with XFGS/XFG (GRS8 RR_Quintile(Q)5vs.Q1_ = 3.82, 95% CI: 1.76, 8.29). GRS8 significantly (*p* = 0.04) improved model prediction from C-index of 88% (95% CI: 0.84, 0.92) to 93% (95% CI: 0.91, 0.95) when added to a basic risk model including age, sex, period at risk, intraocular pressure, and glaucoma family history. Metabolite class analyses revealed positive associations of bile acids and inverse associations of fatty acyls with GRS8 (adjusted *p* < 0.001). Conclusions: XFS GRS8 improved XFGS/XFG prediction, and a higher XFS GRS8 was associated with altered levels of fatty acyl and bile acid metabolite classes.

## 1. Introduction

Exfoliation syndrome (XFS) is an age-related disease where abnormal fibrillar extracellular material accumulates in ocular tissues. XFS can lead to exfoliation glaucoma (XFG), the most common identifiable type of secondary open-angle glaucoma [1]. XFG is associated with a more severe prognosis, increased intraocular pressure, and systemic diseases [2,3]. XFG is an insidious and irreversible disease that has a significant impact on quality of life and for which there are currently no targeted screening strategies or curative treatments, partly due to the limited understanding of the biological mechanisms underlying genetic susceptibility. A genetic risk score (GRS) has shown promise as a screening tool for primary open-angle glaucoma (POAG) [4]. Similarly, the creation of a XFS GRS may enable preliminary evaluations of its potential as a candidate screening tool for XFS/XFG.

Genome-wide association studies (GWAS) have revealed several loci, including lysyl oxidase-like 1 (*LOXL1*), which have been identified as genetic risk factors contributing to the development of XFS [5]. The biological mechanism underlying this genetic susceptibility to XFS has not been well understood. Although it is recognized that *LOXL1* plays an important role in the regulation of extracellular matrix homeostasis, a notable gap remains in our understanding of the systemic effects of the variants [6]. Furthermore, it is noteworthy that a large percentage of the unaffected population (>80%) also possess the same variants [7]. Considering the genetic and environmental heterogeneity inherent in XFS, the development of a XFS GRS that includes *LOXL1* single nucleotide polymorphisms (SNPs) may have clinical utility in identifying individuals who might have a higher risk; however, to the best of our knowledge, such a score has not been evaluated in a prospective population-based study.

Metabolomic profiling may be a promising tool that could advance our understanding of the systemic alterations associated with high genetic susceptibility. Currently, few human metabolomic studies have been completed for XFS/XFG [8,9,10], and none have examined metabolomic profiles in relation to genetic susceptibility among healthy individuals. Although *LOXL1* is the principal genetic determinant of XFS/XFG [11], and plays key roles in elastic fiber renewal and extracellular matrix regulation [12], how *LOXL1*-related molecular perturbations lead to the systemic pathway changes that initiate XFS is poorly understood. Metabolomic analyses may further inform processes contributing to XFS by identifying downstream perturbations in lipid, amino acid, steroid, and other metabolomic pathways. Additionally, large prospective cohorts allow for the evaluation of the clinical utility of GRS for incident disease, reducing susceptibility to selection bias and enhancing generalizability compared to clinic-based studies. Also, a metabolomic study of XFS genetic susceptibility in a large healthy population allows for generating statistically powerful results that can provide potential new insights into the etiology of XFS/XFG. Thus, we generated a XFS GRS incorporating *LOXL1* SNPs, evaluated its clinical utility in US cohorts, and conducted untargeted plasma metabolomics analyses to identify metabolites and metabolite classes associated with high genetic susceptibility to XFS/XFG. We hypothesized that (1) a XFS GRS incorporating *LOXL1* SNPs improves the prediction of XFS/XFG risk, and (2) having a higher XFS GRS is associated with specific metabolites/metabolite classes.

## 2. Materials and Methods

### 2.1. Study Population and Blood Collection

We included participants from three prospective cohorts: Nurses’ Health Study (NHS), Nurses’ Health Study 2 (NHS2), and Health Professional Follow-up Study (HPFS). NHS started in 1976 with 121,700 female nurses aged 30 to 55 years enrolled; NHS2 started in 1989 with 116,429 female nurses aged 25 to 42 years enrolled; and HPFS started in 1986 with 51,529 male health professionals aged 40 to 75 years enrolled. We accounted for baseline year differences by adjusting for age in all models. Detailed descriptions of the cohorts are published elsewhere [13,14]. In all three cohorts, information on lifestyle factors, diet, and medical conditions, including glaucoma, was collected by biennial questionnaires, with follow-up rates exceeding 80%. The study protocol was approved by the institutional review boards of the Brigham and Women’s Hospital, Harvard T.H. Chan School of Public Health, and Icahn School of Medicine at Mount Sinai. This study adhered to the tenets of the Declaration of Helsinki.

For the prospective analyses evaluating metabolite profiles related to GRSs, we followed participants from 1980 to 2018 for the NHS, 1989 to 2019 for the NHS2, and 1986 to 2018 for the HPFS. “Baseline” was defined as the first two-year period in which an eligible participant began contributing person-time. We excluded participants at baseline who: (1) had prevalent cancer (except for nonmelanoma skin cancer), (2) had prevalent glaucoma or glaucoma suspect of any type, (3) died before baseline, or were lost to follow-up within 2 years after baseline, (4) never reported an eye exam during follow-up, (5) had cataract extraction (as detecting exfoliation material may be challenging after lens removal) and (6) had no genetic data. Participants who did not meet the criteria at baseline were included in subsequent follow-up periods if, at later periods, the 6 eligibility criteria were met.

Blood samples were collected from 32,826 NHS participants during 1989–1990; 29,611 NHS2 participants during 1996–1999; 18,159 HPFS participants during 1993–1995. All blood samples were returned to the laboratory via overnight courier and refrigerated before processing. Whole blood samples were centrifuged, and plasma, buffy coat, and red blood cells were aliquoted and stored in liquid nitrogen freezers (≤−130 °C).

### 2.2. GRS and LOXL1 SNPs

A GRS was constructed based on eight SNPs in seven genes (*LOXL1*, *CACNA1A*, *POMP*, *TMEM136*, *AGPAT1*, *SEMA6A* and *RBMS3*) identified as loci for XFS at the genome-wide significant level (*p* < 5 × 10^−8^) from the largest GWAS of XFS, encompassing 9035 XFS cases and 17,008 controls from 24 countries, and replicated in 18 countries with 4803 XFS cases and 93,267 controls [15]. The genotypes were derived from five genotyping platforms: Affymetrix, HumanCoreExome, IlluminaHumanHap, OmniExpress, and OncoArray [16,17]. These genotypes were then imputed using the 1000 Genomes Phase III release dataset [18,19]. Quality control measures for both genotyping and imputation processes were standardized within each platform and across all platforms [16,17].

For GRS calculations, eight genetic variants were coded according to the allele dosage related to a higher risk of XFS, based on imputation score values ranging from zero (no allele), to two (two alleles). The GRS (GRS8) was calculated by standardizing the sum of the weighted products of the effect estimates for each of the eight genetic variants and risk allele dosage across the 8 SNPs [20]. GRS6 was constructed similarly using 6 SNPs (rs12490863, rs10072088, rs4926244, rs7329408, rs11827818, rs3130283) excluding the *LOXL1* SNPs. To better understand associations with GRS8, we also constructed GRS2 using the two *LOXL1* SNPs (rs3825942, rs1048661) to compare the contributions of GRS6 versus GRS2. Details of genotyping and GRS calculations are described in the Supplementary Materials.

### 2.3. XFG Suspect (XFGS)/XFG Ascertainment

We were interested in factors contributing to ocular exfoliation material formation; however, because we asked participants about glaucoma diagnoses rather than XFS specifically, we defined our primary outcome as XFGS/XFG to capture individuals with XFS most likely to report a glaucoma diagnosis. Among participants at risk of glaucoma who were followed from baseline until 2018 (NHS), 2019 (NHS2), and 2018 (HPFS), XFGS/XFG cases were identified. In the first step of case confirmation, we identified participants who self-reported a new physician diagnosis of glaucoma from biennial questionnaires. Second, we collected medical records from the diagnosing eye care provider to confirm information on the presence of exfoliation material or other secondary causes for elevated intraocular pressure (IOP), maximum untreated IOP, optic nerve features, status of filtration apparatus, ophthalmic surgery, and earliest visual field (VF) loss date. Third, all records were reviewed using standardized criteria by a glaucoma specialist (L.R.P.). XFGS/XFG cases were defined as the presence of exfoliation material in either eye along with (1) maximum untreated IOP ≥ 22 mmHg or (2) cup-to-disc ratio ≥ 0.6 or an inter-eye difference in cup-to-disc ratio ≥ 0.2 or (3) glaucomatous VF loss on reliable VF tests. Participants with XFS only, those whose medical records could not be obtained, or those diagnosed with other types of glaucoma were censored during follow-up to minimize detection bias. All three cohorts used the same disease confirmation process and definition.

### 2.4. Metabolomic Profiling

Metabolomic profiling of high XFS genetic susceptibility was conducted in a subset of the NHS, NHS2, and HPFS participants (n = 7547) who had pre-diagnostic plasma metabolites and genetic data available. Plasma metabolites using liquid chromatography-tandem mass spectrometry were measured at the Broad Institute of MIT and Harvard University (Cambridge, MA, USA) [21]. A pilot study for quality control was used to assess the stability of the metabolites of immediate processing vs. 24-h to 48-h delayed processing. Among a total of 642 measured known metabolites, 127 metabolites failed a quality control pilot study to evaluate stability with processing delays, which is of critical importance given our sample collection methods [21], and 88 metabolites were excluded because analytic models did not converge. Most of the latter type of metabolites were xenobiotics with detectable levels in only a few participants, resulting in insufficient power for evaluation. After these exclusions, we included 427 chemically identified metabolites in our analysis. We imputed metabolites with a frequency of missing values being <25% in the study population by using values that represented half of the minimum value for that metabolite [22,23]. Metabolites were standardized to probit scores for analyses on a common scale. All metabolites had good within-person stability over 1–2 years [21]. Details of metabolomic profiling are described in the Supplementary Materials.

### 2.5. Covariates

In analyses of the relation between GRS and incident XFGS/XFG, we adjusted for potential XFG risk factors including age (years), ancestry (Scandinavian European, southern European, other European, other races), glaucoma family history (yes/no), body mass index (kg/m^2^), self-reported history (yes/no) of hypertension, diabetes mellitus, hypercholesterolemia, or myocardial infarction; cigarette smoking (pack-years), alcohol intake (g/day), caffeine intake (mg/day), folate intake (µg/day), vitamin A intake (IU/day), total calories intake (kcal/day), Alternate Healthy Eating Index-2010 excluding alcohol intake (0–100 score), UV flux (mW/m^2^), weighted lifetime average latitude of residence (°N), and physical activity (metabolic-equivalents of task-hour/week). For evaluating the clinical utility of GRSs and SNPs for XFGS/XFG, we adjusted for age, sex, period at risk, age and sex interaction, elevated IOP > 25 mmHg, and family history of glaucoma. For metabolomics analyses of XFS genetic susceptibility, in the main analyses, we adjusted for age, sex, fasting status (fasting more than 8 h before blood draw, yes/no), and top 10 principal components derived from each genotyping platform to account for potential population stratification and technical variation [24]. In secondary analyses, we further adjusted for the covariates described above for the prospective analyses of the XFS GRS and XFGS/XFG risk.

### 2.6. Statistical Analysis

#### 2.6.1. Clinical Utility of XFS GRS

To test the clinical utility of the GRSs in predicting XFGS/XFG, we conducted per-person multivariable Cox proportional hazards regression models to calculate estimates of multivariable-adjusted relative risks (RRs) and corresponding 95% confidence intervals (CIs) of incident XFGS/XFG in association with GRS quintiles. We combined the 1st and 2nd quintiles of GRS in the models to maximize statistical power (given the few cases in these groups), and the 3rd quintile was used as the reference group [4]. Inverse probability weighting was applied to the three cohorts to account for potential selection bias due to the selection process into various GWAS studies that generated the genetic data. Data from participants were weighted by the inverse of their probability of being selected into GWAS [25]. Details of inverse probability weighting were presented in the Supplementary Materials. We also estimated the *LOXL1* SNPs rs3825942_G allele_, and rs1048661_G allele_ in relation to XFGS/XFG using Cox regression models as the betas for these *LOXL1* SNPs are much larger than the other GRS8 SNPs. Because there were few incident XFGS/XFG cases among participants with AA and AG genotypes for the rs3825942 SNP, we combined these two groups in the models.

The Harrell’s C-statistic (i.e., concordance index), which accounts for time-dependent variables, was calculated using Cox proportional hazards models as an approximate equivalent of the receiver operating characteristic (ROC) under the curve (AUC). We included GRS2 and GRS6 in the models to have a better comparison of the associations with the *LOXL1* SNPs versus the remaining 6 SNPs in predicting XFGS/XFG. Additionally, to evaluate whether the addition of GRSs significantly improved model prediction, we compared Harrell’s C-statistics using the DeLong test. All significance tests were 2-sided at a significance level α = 0.05. Analyses were conducted with SAS (Version 9.4, SAS Institute, Cary, NC, USA).

#### 2.6.2. Metabolomic Analysis of High XFS Genetic Susceptibility

We conducted metabolomics analyses as the second part of our study among participants who had such data available (n = 7547). For individual metabolite analyses, we used linear regression models to estimate the mean differences in metabolite levels by each GRS (per 1 standard unit increase) and SNPs status (per risk allele increase). To account for multiple comparisons for correlated metabolites, we used the number of effective tests (NEF) [26], with NEF < 0.05 considered statistically significant, and NEF < 0.2 considered as worthy of further investigations.

Metabolite set enrichment analysis (MSEA) [27] was used to evaluate the associations of GRS and SNPs with metabolite classes (n = 20). We assigned a metabolite class based on chemical taxonomy for 306 of the 427 metabolites. In total, there were 20 metabolite classes with at least 3 metabolites per class: bile acids; lysophosphatidylcholines; phosphatidylcholine plasmalogens; cholesteryl esters; sphingomyelins; lysophosphatidylethanolamines; phosphatidylcholines; organoheterocyclic compounds; triglycerides with <3 double bonds (DB); organic acids and derivatives; PE plasmalogens; carboxylic acids and derivatives; triglycerides with ≥3 DB; phosphatidylethanolamines; carnitines; nucleosides, nucleotides, and analogues; ceramides; diglycerides; fatty acyls; and phosphatidylserines. False discovery rate (FDR) was used to control for multiple comparisons [28]. An FDR < 0.05 was considered statistically significant, and an FDR < 0.2 was considered as worthy of further investigation. Metabolite analyses were conducted in R v4.2.0 (R Foundation for Statistical Computing).

## 3. Results

### 3.1. Clinical Characteristics of XFGS/XFG Cases

Characteristics of XFGS/XFG cases vs. non-cases are shown in Table 1. XFGS/XFG cases were generally older (54.3 vs. 49.3 years), had fewer comorbidities (e.g., self-reported diabetes 0% vs. 2.8%; self-reported hypertension 16.3% vs. 17.7%), and a higher GRS8 (0.841 vs. −0.002) compared with non-cases. The characteristics of the study population subset with metabolomics data available by XFS GRS8 tertiles (n = 7547) are shown in Table S1. The age at blood draw was similar across each XFS GRS8 tertile, averaging around 56 years. Tertile 3 had the greatest percentage of participants with positive glaucoma family history (16.3% vs. 15.8% vs. 14.7% for T3, T2, and T1, respectively), diabetes (4.3% vs. 3.4% vs. 3.6% for T3, T2, and T1, respectively), and hypertension (26.0% vs. 24.2% vs. 24.6% for T3, T2, and T1, respectively).

The clinical characteristics of incident XFGS/XFG cases (n = 118) by XFS GRS are shown in Table 2. The mean age at diagnosis was similar across all tertiles at 70–71 years. T3 had the highest untreated intraocular pressure (mean = 28.2) and cup to disc ratio (mean = 0.59). Additionally, we observed the lowest percentages of family history of glaucoma (9.2%), bilaterality (32.6%), and visual field loss (40.3%) in T3. The lowest percentages for bilaterality and visual field loss may reflect earlier detection and treatment among individuals who have a high genetic risk for XFS. The lower prevalence of family history of glaucoma may be reflective of the fact that family history in general reflects proportionately more primary open-angle glaucoma history than XFG history, and thus in T3, we may have observed a lower prevalence.

### 3.2. Association Between XFS GRS8, GRS6, GRS2, LOXL1 SNPs and Incident XFGS/XFG

Participants in the highest quintile of GRS8 had significantly higher XFGS/XFG risk (Table 3), with a relative risk (RR) of 3.82 (95% CI: 1.76, 8.29) compared with participants of average genetic risk (Q3). The RR for being in the first two quintiles combined was 0.13 (95% CI: 0.04, 0.48) compared to Q3. After adjusting for covariates, the RR increased to 4.30 (95% CI: 2.15, 8.59) for the highest quintile, compared with Q3. The inclusion of elevated IOP (model 3) further increased the RR of XFGS/XFG for participants in Q5 to 7.03 (95% CI: 3.07, 16.09) compared with Q3. Adding family history of glaucoma to the model (model 4) did not substantially affect the estimate (Q5 vs. Q3, RR = 4.31 (95% CI: 2.12, 8.76)).

*LOXL1* SNP rs1048661_G allele_ GG alleles were significantly associated with an increased risk of XFGS/XFG compared with TT alleles (RR = 3.82 (95% CI: 1.09, 13.45)) (Table 4a). Similar results were observed after adjusting for covariates (model 2) (GG vs. TT, RR = 4.47 (95% CI: 1.22, 16.31)). The RR increased after adjusting for elevated IOP (model 3, GG vs. TT, RR = 8.53 (95% CI: 3.02, 24.15)), and family history of glaucoma (model 4, GG vs. TT, RR = 4.62 (95% CI: 1.25, 17.06)), respectively. Results were similar for *LOXL1* SNP rs3825942; participants with GG risk alleles had a higher risk of XFGS/XFG compared with participants with AA and AG combined (GG vs. AA + AG, RR = 70.65 (95% CI: 17.18, 290.52)).

We examined the associations between GRS6 (constructed using 6 non-*LOXL1* SNPs) and XFGS/XFG (Table 4b) and found no significant associations across all models. We then evaluated the associations of GRS2 (constructed using 2 *LOXL1* SNPs) with XFGS/XFG, as shown in Table 4c. GRS2 Q4 (RR = 2.06 (95% CI: 1.03, 4.11)) and Q5 (RR = 3.47 (95% CI: 1.82, 6.61)) were significantly associated with increased risk of XFGS/XFG. These associations remained significant after adjusting for covariates in model 2 (e.g., RR_Q5vs.Q3_ = 3.70 (95% CI: 1.93, 7.12)), further adjusting for IOP in model 3 (e.g., RR_Q5vs.Q3_ = 4.99 (95% CI: 2.62, 9.53)), and additionally family history of glaucoma in model 4 (e.g., RR_Q5vs.Q3_ = 3.67 (95% CI: 1.90, 7.08)). Together, these results suggest that the strong associations observed for GRS8 are primarily driven by the *LOXL1* SNPs.

### 3.3. Model Prediction Performance for XFGS/XFG

The univariate Cox proportional hazards regression model for GRS8 yielded a concordance of 0.76 (95% CI: 0.72, 0.79) for predicting XFGS/XFG (Table 5). Given the strong established associations between *LOXL1* SNPs and XFGS/XFG, we separately evaluated GRS2, the two *LOXL1* SNPs, and GRS6. The univariate model for GRS2 (model 1b) achieved a concordance of 0.73 (95% CI: 0.70, 0.77). Including both *LOXL1* SNPs in a univariate model (model 1e) increased concordance to 0.76 (95% CI: 0.72, 0.79). GRS6 alone (model 1f) showed low predictive ability (C-index = 0.51; 95% CI: 0.46, 0.57), but adding both *LOXL1* SNPs (model 1g) increased concordance to 0.76 (95% CI: 0.73, 0.80)—the concordance was better than the model 1b with GRS2 alone, suggesting some improvement in the model prediction when GRS6 was included.

In a multivariable model adjusted for age, sex, elevated IOP, and family history, the concordance was 0.88 (95% CI: 0.84, 0.92). Adding GRS8 to this model significantly increased concordance to 0.93 (95% CI: 0.91, 0.95; *p* = 0.04). In models 5b and 5c, separately adding the *LOXL1* SNPs rs3825942 or rs1048661 resulted in non-significant increases in the C-statistic to 0.91 (95% CI: 0.89, 0.93; *p* = 0.31) and 0.90 (95% CI: 0.88, 0.92; *p* = 0.37), respectively. Furthermore, model 5d (adding both *LOXL1* SNPs) and model 5e (adding GRS2) to the base multivariable model (model 4) produced results equivalent to model 5a (adding GRS8). This indicates that the high predictive performance of GRS8 was largely attributable to the effects of the *LOXL1* SNPs. Additionally, we conducted sensitivity analyses excluding 60 XFGS cases (Table S2a) and excluding 58 XFG cases (Table S2b), which yielded similar results.

### 3.4. Associations of Individual Metabolites and GRS and SNPs

**Figure 1:**
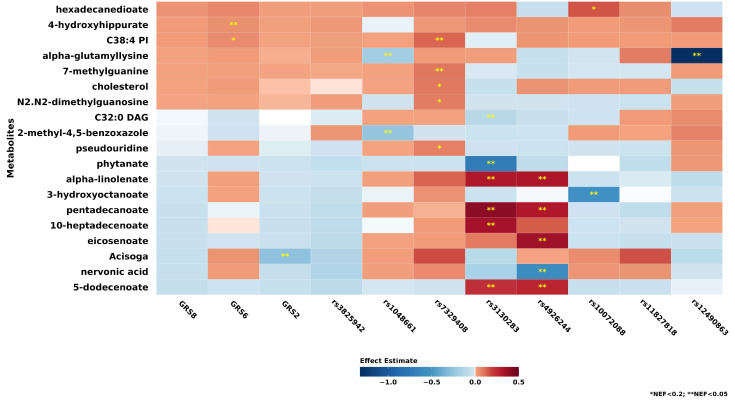
19 individual metabolites among 427 metabolites evaluated that were NEF < 0.2 for the linear regression model (n = 7547) for GRS8, GRS6, GRS2, and 8 SNPs, ordered by strength of effect estimates for GRS8. All models adjusted for age, fasting status, top 10 principal components. * NEF < 0.2; ** NEF < 0.05. Abbreviations: PI, phosphatidylinositol; DAG, diacylglycerol; GRS8, genetic risk score constructed using 8 single nucleotide polymorphisms (SNPs) (rs1048661, rs3825942, rs12490863, rs10072088, rs4926244, rs7329408, rs11827818, rs3130283); GRS6, genetic risk score constructed using 6 SNPs (rs12490863, rs10072088, rs4926244, rs7329408, rs11827818, rs3130283), excluding the two *LOXL1* SNPs rs1048661, rs3825942; GRS2, genetic risk score constructed using the two *LOXL1* SNPs rs1048661, rs3825942; NEF, number of effective tests corrected *p*-value.

Although the primary exposure was GRS8, given the hypothesis-generating nature of the study, we investigated the metabolomics profiles for GRS8, GRS6, GRS2, as well as each component SNP of GRS8. Figure 1 shows the individual metabolites associated (NEF < 0.2) with GRS8, GRS6, GRS2, and the 8 SNPs’ linear regression models among 7547 participants who had metabolite data available (the associations with the 427 individual metabolites are provided in Table S3). Among 427 measured metabolites, 15 metabolites were NEF-significant with GRS2, GRS6, or the 6 individual SNPs. However, no significant associations were observed for GRS8, and the *LOXL1* SNP rs3825942_G allele_. The strongest association was found between rs12490863_A allele_ and alpha-glutamyllysine (mean difference = −1.31 (95% CI: −1.40, −1.23); NEF < 0.001), which was also significantly and inversely associated with the *LOXL1* SNP rs1048661_G allele_ (mean difference = −0.19 (95% CI: −0.25, −0.12); NEF < 0.001). Acisoga was NEF-significantly associated with GRS2 (mean difference = −0.27 (95% CI: −0.34, −0.20); NEF < 0.001), but not with GRS8, or the 2 individual *LOXL1* SNPs.

In secondary analyses adjusting for covariates that may affect metabolite levels or XFGS/XFG risk (Figure S1), taurochenodeoxycholic acid showed NEF-significant associations with GRS8 (mean difference = 0.12 (95% CI: 0.06, 0.18); NEF = 0.03) and GRS2 (mean difference = 0.12 (95% CI: 0.06, 0.18); NEF = 0.03).

### 3.5. Associations of Metabolite Classes and GRS and SNPs

**Figure 2:**
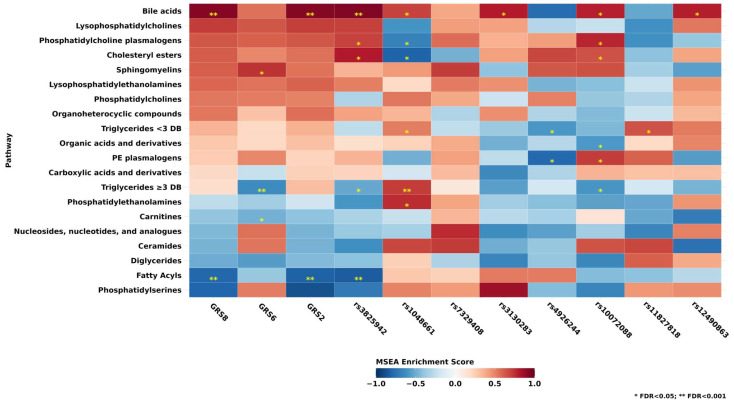
Metabolite classes (n = 20) evaluated in the adjusted linear regression models (n = 7547) for GRS8, GRS6, GRS2, and SNPs. All models adjusted for age, fasting status, and top 10 principal components. * FDR < 0.05; ** FDR < 0.001. Abbreviations: DB, double bond; PE, phosphatidylethanolamine; GRS8, genetic risk score constructed using 8 single nucleotide polymorphisms (SNPs) (rs1048661, rs3825942, rs12490863, rs10072088, rs4926244, rs7329408, rs11827818, rs3130283); GRS6, genetic risk score constructed using 6 SNPs (rs12490863, rs10072088, rs4926244, rs7329408, rs11827818, rs3130283), excluding the two *LOXL1* SNPs rs1048661, rs3825942; GRS2, genetic risk score constructed using the two *LOXL1* SNPs rs1048661, rs3825942; MSEA, metabolite set enrichment analysis; FDR, false discovery rate.

For metabolite set enrichment analysis among 20 metabolite classes (Figure 2) in relation to GRS or SNPs, we found 11 metabolite classes that were FDR < 0.05, and 3 metabolite classes that were FDR < 0.001: fatty acyls, triglycerides with ≥3 double bonds, and bile acids (the associations with the metabolite classes are provided in Table S4). Bile acids were positively associated with GRS8 (Enrichment Score (ES) = 0.91; FDR < 0.001), GRS2 (ES = 0.92; FDR < 0.001) and *LOXL1* SNP rs3825942_G allele_ (ES = 0.93; FDR < 0.001). Associations of the individual metabolites included in the bile acids class are shown in Figure S2. Fatty acyls showed inverse associations with GRS8, GRS2, and *LOXL1* SNP rs3825942_G allele_ (FDR < 0.001). Figure S3 shows the associations of the individual metabolites included in the fatty acyls class, with the majority of the associations being inverse for GRS8 and the *LOXL1* SNP rs3825942_G allele_, including eicosapentaenoic acid (EPA) (GRS8: mean difference = −0.052 (95% CI: −0.11, 0.0057)), docosahexaenoic acid (DHA) (GRS8: mean difference = −0.048 (95% CI: −0.11, 0.0094)) and also oleic acid (GRS8: mean difference = −0.048 (95% CI: −0.11, 0.0094)), although these individual associations were not significant (NEF > 0.2). Triglycerides with ≥3 double bonds showed significant inverse associations with GRS6 (FDR < 0.001) and positive associations with *LOXL1* SNP rs1048661 (FDR < 0.001). Individual associations within the triglycerides containing ≥3 DBs are shown in Figure S4.

Additionally, among metabolites that were not included in MSEA analyses as there were <3 metabolites to constitute a class, we observed non-significant inverse associations between GRS8 and cortisol (GRS8: mean difference = −0.0046 (95% CI: −0.028, 0.019)) and cortisone (GRS8: mean difference = −0.023 (95% CI: −0.046, 0.00019)), which are non-bile acid steroids. Interestingly, cortisone was previously also significantly inversely associated with XFGS/XFG in a previous publication [8].

In secondary analyses, we further conducted metabolite set enrichment analyses using models with additional adjustment for covariates (Figure S5); the results remained similar compared with those in Figure 2, with fatty acyls and bile acids being significantly associated with GRS8, GRS2, and *LOXL1* SNP rs3825942_G allele_.

### 3.6. Subgroup Analyses

We evaluated whether our results varied by subgroups defined by age (Figure S6), sex (Figure S7), and latitude (Figure S8). Overall, results remained similar across all subgroups, with no significant interactions observed by these stratification factors. The associations with alpha-glutamyllysine were suggestively different by age groups: we observed positive associations in those aged ≤56 years and inverse associations in those aged >56 years. Also, stronger inverse associations were found among participants living below 41° N for fatty acyls (oleic acid and heptadecanoic acid) and GRS8.

## 4. Discussion

We demonstrated that incorporating 8 SNPs from the largest XFS GWAS [15], including *LOXL1* variants, resulted in modest improvements in risk prediction of XFGS/XFG compared to a base model containing age, sex, IOP, and glaucoma family history. Notably, *LOXL1* SNPs contributed the most to the predictive power of the GRS. Subsequently, we examined the metabolomic profiles associated with high GRS8, GRS6, GRS2 and the 8 SNPs individually. Our study found that GRS8, GRS2, and the *LOXL1* SNP rs3825942_G allele_ were positively associated with bile acids and inversely associated with fatty acyls. These associations warrant further investigation in future studies.

### 4.1. LOXL1 and XFS GRS

Our findings on the predictive performance of the XFS GRS in our population-based study support its potential utility for early detection of XFGS/XFG. The strong performance of the GRS—particularly when *LOXL1* SNPs are included—is consistent with previous studies identifying *LOXL1* as a major genetic determinant of XFS/XFG across different populations [11,29,30,31]. *LOXL1* belongs to the lysyl oxidase gene family, a group of enzymes that synthesize and maintain elastic fibers [32]. *LOXL1* plays a key role in renewing the elastic tissues and maintaining proper extracellular matrix structure [12], which might affect aqueous humor outflow, elevate IOP, and result in glaucomatous damage [33]. Meta-analysis of *LOXL1* polymorphisms and XFS/XFG have also found significant associations. However, there were some inconsistencies observed in different populations. For example, rs1048661 G allele is not significantly associated with XFS/XFG in Chinese [34] and Japanese populations [35], and the rs3825942 G allele is not associated with XFS/XFG in a South African population.

Although limited research has directly assessed the relation between XFS genetic risk score and glaucoma, recent studies have applied POAG PRS to stratify XFG severity [36]. We observed no differences in age at diagnosis across XFS GRS tertiles. However, IOP and cup-to-disc ratio were highest among participants in the top XFS GRS tertile. Interestingly, the top tertile also had the lowest proportion of bilateral XFGS/XFG and glaucomatous visual field loss at diagnosis, which may suggest that individuals with higher genetic risk are detected and treated earlier, likely due to higher IOP and cup-to-disc ratios.

XFG often presents at an advanced stage and progresses rapidly once diagnosed. Thus, early detection of at-risk individuals could have substantial clinical benefits. Although the disease prevalence of XFG varies among different populations, considering the high risk of conversion from XFS to XFG, as well as the poorer visual prognosis, targeted screening strategies for genetically high-risk individuals warrant further investigations. Population-wide screening for XFS/XFG is unlikely to be cost-effective in low-prevalence settings, especially given that a large percentage of the unaffected population (>80%) also possess some of the same variants [7]. However, incorporating GRS into a two-tier screening strategy could improve efficiency by enriching the pool of individuals undergoing detailed ophthalmic examination. For example, in our study, individuals in the highest quintile of the XFS GRS had a 3.82-fold higher risk of disease compared with those in the middle quintile. If a first-tier genetic screen were used to identify individuals in this high-GRS group, subsequent targeted ophthalmic evaluation could substantially increase the positive predictive value of detection. However, clinical implementation of GRS as a screening tool requires prospective clinical evaluation of participants recalled for ocular phenotyping based on their GRS profile combined with a cost-effectiveness analysis.

### 4.2. Metabolomic Profiles of XFS GRS and SNPs Status

A previous health professionals study assessing the metabolomic profile of XFGS/XFG found that cortisone and triglycerides were inversely associated, whereas lysophosphatidylcholines and phosphatidylethanolamine plasmalogens showed positive associations [8]. In this study, we observed a similar inverse association between cortisone and both the XFS GRS and *LOXL1* SNPs (Figure S2), although these associations did not reach statistical significance. Cortisol is a glucocorticoid, a potent anti-inflammatory hormone that stabilizes vascular and tissue barriers. Insufficient endogenous glucocorticoid levels could fail to suppress inflammation, permit breakdown of the uveal blood-ocular barrier, and allow plasma proteins and matrix components to leak into the anterior segment to form exfoliation material [37]. Additionally, relatively low cortisol levels may be a marker of high exposure to oxidative stressors, as some studies have suggested that low cortisol levels may be linked to greater UV exposure [38], which is a known environmental risk factor for XFS/XFG [39]. We found that triglycerides with ≥3 double bonds were inversely associated with GRS6, as well as rs10072088_T allele_ and *LOXL1* SNP rs3825942_G allele_ and positively associated with *LOXL1* rs1048661_G allele_. In contrast, triglycerides with <3 double bonds showed significant positive associations with rs11827818_G allele_ and *LOXL1* rs1048661_G allele_. Elevated levels of triglycerides with ≥3 double bonds have been linked to reduced risk of cardiovascular events, potentially reflecting lower hyperglycemia levels, which may be associated with exfoliation material accumulation in the eye [40]. Additionally, another health professionals study using pre-diagnostic metabolomic profiles to identify XFGS/XFG endotypes reported that compared to the most common endotype 2, triglycerides with ≤3 double bonds were significantly lower in endotype 1 (characterized by physically active, metabolically healthy males with higher UV exposure), and higher in endotype 3 (characterized by low genetic susceptibility to XFS/XFG, lower physical activity, higher BMI, and greater systemic metabolic dysfunction) [41]. These mixed findings highlight the complex etiology of XFS/XFG and underscore the need for future studies to explore the interplay between triglyceride subtypes, genetic risk, and environmental factors. In our study, we did not observe significant associations between the XFS GRS or *LOXL1* SNPs and lysophosphatidylcholines or phosphatidylethanolamine plasmalogens. This may suggest that these two metabolite classes are more strongly influenced by environmental factors rather than genetic factors.

*Bile acids.* We found significant positive associations between bile acids with GRS8, GRS2, and *LOXL1* SNPs rs3825942 and rs1048661. Previous studies have shown that serum bile acid levels are elevated in patients with POAG, implicating bile acids in ocular oxidative stress and cell death [42]. Bile acids can also activate profibrotic pathways that promote epithelial–mesenchymal transition and myofibroblast differentiation, aligning with XFS pathology characterized by excessive extracellular matrix deposition [43], a process driven in part by *LOXL1*-mediated extracellular matrix formation and remodeling. Additionally, bile acids can signal immune cells to trigger inflammatory cascades, and cytokine release [44]. Patients with XFS/XFG were found to have elevated levels of inflammatory markers [45], which may suggest that elevated plasma bile acids may exacerbate the inflammation. Together, these observations suggest genetic susceptibility to XFS/XFG through *LOXL1*-linked extracellular matrix and fibrotic changes interacts with bile acid signaling to promote oxidative stress, fibrosis, and inflammation in XFS/XFG.

*Fatty acyls.* We found significant inverse associations of fatty acyls and GRS8, GRS2, and *LOXL1* SNP rs3825942_G allele_, which suggests that fatty acyls may be linked to a lower genetic susceptibility of XFS, and indicate a potential protective role in XFGS/XFG pathogenesis. Previous studies have reported protective effects of fatty acyls and glaucoma. One cross-sectional study found dietary intake of fatty acid was associated with a lower risk of glaucoma [46]. Another randomized controlled trial among XFG patients found that oral supplementation of DHA/EPA with antioxidants for half a year led to significantly lower oxidative stress, and lowered IOP compared with baseline [47]. Mechanistically, fatty acyls have antioxidant and anti-inflammatory properties, and may beneficially modulate IOP regulation and neuroprotection pathways that could counteract the oxidative stress, inflammation, and thus may play a protective role in XFS/XFG pathogenesis [48]. In our study, DHA and EPA showed non-significant inverse associations with XFS GRSs and *LOXL1* SNP rs3825942_G allele_ (Figure S3) at the individual metabolite level, but the overall fatty acyl class-level inverse association was statistically significant, which warrants further investigations.

### 4.3. Strengths and Limitations

Our study had several strengths. For the GRS clinical utility study, we had a large overall study population (n = 39,472) with 730,855 person-years of follow-up. We also had extensive covariate data available, including lifestyle factors, known XFGS/XFG risk factors, and detailed clinical characteristics, allowing for a more comprehensive analysis. The metabolomics profiling analyses included a large population (n = 7547) and covered a wide range of metabolites (n = 427), which enhanced our ability to detect novel associations.

However, several limitations should be acknowledged. The study population primarily consisted of highly educated health professionals, with most participants being of European ancestry and a high proportion being female. These demographic characteristics may limit the generalizability of our findings. Furthermore, the associations between genetic risk and metabolite levels were cross-sectional, as we lacked repeated metabolomic measurements over time during follow-up. Additionally, these associations may be overestimated due to a lack of external validations. Future studies, including clinical translation efforts, are needed to validate these findings in racially, ethnically, and socioeconomically diverse cohorts. Lastly, although long-term sample storage may raise concerns about potential degradation or interaction of metabolites, previous studies [49,50] using the same NHS, NHS2, and HPFS cohort samples have demonstrated reproducibility and consistency across different cohorts [51,52,53], providing reassurance that such issues likely had minimal impact on our results.

In conclusion, we observed that a XFS GRS incorporating 8 SNPs from the largest XFS GWAS, including *LOXL1* variants, modestly but significantly improved model prediction of XFGS/XFG cases compared to a basic nongenetic model. GRS8, GRS2, and the *LOXL1* SNP rs3825942_G allele_ were positively associated with bile acids and inversely associated with fatty acyls. We addressed an important gap in the current literature, where limited research has explored the predictive utility of the XFS GRS for early identification of XFGS/XFG in a population-based study. Also, our work contributes to the evidence for metabolomic profiles associated with high genetic susceptibility, offering insights into the biological pathways involved in XFS/XFG development. Given the exploratory nature of these findings, future independent studies including more diverse populations are needed to validate and expand upon our results.

## Figures and Tables

**Table 1:** Age and age-adjusted characteristics of incident XFGS/XFG cases vs. non-cases from NHS (1980–2018), NHS2 (1989–2019), and HPFS (1986–2018) at study entry.

Characteristics ^a^	Incident XFGS/XFG Cases (n = 118)	Non-Cases (n = 39,354)	*p*-Values
Genetic risk score-8	0.841 (0.461)	−0.002 (0.999)	<0.001
Female, %	79.6	74.1	<0.001
Age, years ^b^	54.3 (8.4)	49.3 (8.9)	0.001
Scandinavian ancestry, %	11.9	9.3	0.80
Family history of glaucoma, %,^b^	26.2	16.3	0.01
Self-reported diabetes diagnosis, %	0	2.8	0.02
Self-reported hypertension diagnosis, %	16.3	17.7	0.43
Self-reported high cholesterol diagnosis, %	5.4	17.4	<0.001
Self-reported history of myocardial infarction, %	0	1.5	0.04
Total calories, kcal/day	1631 (580)	1779 (521)	0.01
Total vitamin A intake, IU/day	13,501 (6011)	13,519 (8113)	0.33
Total caffeine intake, mg/day	384 (265)	291 (237)	<0.001
Folate intake, μg/day	434 (221)	44 (243)	0.26
Weighted lifetime average latitude of residence, °N	40.6 (3.6)	39.5 (3.9)	0.01
Annual UV flux, ×10^−4^ mW/m^2^	181 (18)	186 (27)	0.03
Total alcoholic intake, g/day	9.0 (16.3)	6.8 (11.2)	0.27
Cigarette smoking, pack-years	7.0 (12.5)	9.2 (15.1)	0.04
Body mass index, kg/m^2^	23.4 (3.1)	24.8 (4.1)	<0.001
In top 25th percentile for physical activity, %	14.5	23.9	0.53
Alternate healthy eating index 2010	44.6 (9.8)	45.3 (9.7)	0.48

Abbreviations: XFGS, exfoliation glaucoma suspect; XFG, exfoliation glaucoma; SD, standard deviation; UV, ultraviolet. ^a^ Values are presented as means (SD) for continuous variables and percentages for categorical variables. ^b^ All values other than age have been directly standardized to age distribution of all the participants.

**Table 2:** Clinical characteristics of incident XFGS/XFG cases (n = 118) by XFS GRS8 tertiles *.

Variables	T1 (n = 38)	T2 (n = 38)	T3 (n = 42)
XFS GRS8, median (min, max)	0.408 (−1.020, 0.793)	0.946 (0.808, 1.075)	1.183 (1.076, 1.731)
Age at diagnosis, mean year	70.5	70.3	70.3
Bilateral, %	35.7	44.1	32.6
Highest untreated intraocular pressure, mean mmHg	27.8	27.7	28.2
Cup to disc ratio, mean	0.56	0.55	0.59
Presence of glaucomatous visual field loss, %	46.6	61.5	40.3
Family history of glaucoma, %	22.4	35.8	9.2

Abbreviations: XFGS, exfoliation glaucoma suspect; XFG, exfoliation glaucoma; XFS, exfoliation syndrome; GRS8, genetic risk score constructed using 8 single nucleotide polymorphisms (rs1048661, rs3825942, rs12490863, rs10072088, rs4926244, rs7329408, rs11827818, rs3130283); T, tertile. * Adjusted for age, ancestry (Scandinavian Caucasian, southern European Caucasian, other Caucasian, other races), self-reported history (yes/no) of hypercholesterolemia, hypertension, diabetes, and myocardial infarction, body mass index (kg/m^2^; <22, 22–23, 24–25, 26–27, 28–29, 30+), averaged intakes of total energy (kcal/day), caffeine (mg/day), folate (μg/day), and vitamin A (IU/day); pack-years of smoking (pack-years), alternate health eating index-2010, UV flux (Robertson-Berger units, quintiles), latitude (°N, quintiles), physical activity (metabolic-equivalents of task-hour/week, quartiles).

**Table 3:** Multivariable adjusted relative risks (95% confidence intervals) of XFGS/XFG by quintiles of the XFS GRS8 in per-person Cox regression models.

	Q1 + Q2	Q3	Q4	Q5
Median GRS8 (min, max)	−0.91 (−3.25, 0.02)	0.24 (0.03, 0.40)	0.63 (0.40, 0.95)	1.13 (0.95, 1.89)
XFGS/XFG cases (n = 118)	5	13	41	59
Person-years (%)	292,345 (40.0)	146,095 (20.0)	146,257 (20.0)	146,158 (20.0)
Model 1	0.13 (0.04, 0.48)	REF = 1.0	1.78 (0.78, 4.06)	3.82 (1.76, 8.29)
Model 2	0.13 (0.04, 0.48)	REF = 1.0	2.36 (1.15, 4.85)	4.30 (2.15, 8.59)
Model 3	0.18 (0.05, 0.62)	REF = 1.0	4.29 (1.78, 10.36)	7.03 (3.07, 16.09)
Model 4	0.13 (0.04, 0.51)	REF = 1.0	2.29 (1.11, 4.74)	4.31 (2.12, 8.76)

Abbreviations: XFGS, exfoliation glaucoma suspect; XFG, exfoliation glaucoma; XFS, exfoliation syndrome; GRS8, genetic risk score constructed using 8 single nucleotide polymorphisms (rs1048661, rs3825942, rs12490863, rs10072088, rs4926244, rs7329408, rs11827818, rs3130283); Q, quintile; REF: reference group. Model 1: stratified by age in months, sex, period at risk. Model 2: stratified by age in months, sex, period at risk, and adjusted for the following variables: ancestry (Scandinavian Caucasian, southern European Caucasian, other Caucasian, other races), self-reported history (yes/no) of hypercholesterolemia, hypertension, diabetes, and myocardial infarction; body mass index (kg/m^2^; <22, 22–23, 24–25, 26–27, 28–29, 30+), averaged intakes of total energy (kcal/day), caffeine (mg/day), folate (µg/day), and vitamin A (IU/day); pack-years of smoking (pack-years), alternate healthy eating index-2010, UV reflux (Robertson-Berger units, quintiles), latitude (°N, quintiles), physical activity (metabolic-equivalents of task-hour/week, quartiles). All adjusted covariates were included at baseline. Model 3: Model 2 + intraocular pressure > 25 mmHg. Model 4: Model 2 + family history of glaucoma.

**Table 4:** (**a**). Multivariable adjusted relative risks (95% confidence intervals) of XFGS/XFG by *LOXL1* SNPs in per-person Cox regression models. (**b**). Multivariable adjusted relative risks (95% confidence intervals) of XFGS/XFG by quintiles of the XFS GRS6 in per-person Cox regression models. (**c**). Multivariable adjusted relative risks (95% confidence intervals) of XFGS/XFG by quintiles of the XFS GRS2 in per-person Cox regression models.

**(a)**				
**rs1048661 allele**	**TT**	**TG**	**GG**	
XFGS/XFG cases (n = 118)	4	29	85	
Person-years (%)	75,918 (10.4)	320,648 (43.9)	334,289 (45.7)	
Model 1	REF = 1.0	1.51 (0.39, 5.77)	3.82 (1.09, 13.45)	
Model 2	REF = 1.0	1.65 (0.42, 6.52)	4.47 (1.22, 16.31)	
Model 3	REF = 1.0	2.17 (0.71, 6.63)	8.53 (3.02, 24.15)	
Model 4	REF = 1.0	1.68 (0.41, 6.81)	4.62 (1.25, 17.06)	
**rs3825942 allele**	**AA + AG**		**GG**	
XFGS/XFG cases (n = 118)	2		116	
Person-years (%)	216,204 (29.6)		514,651 (70.4)	
Model 1	REF = 1.0		70.65 (17.18, 290.52)	
Model 2	REF = 1.0		99.72 (19.22, 517.26)	
Model 3	REF = 1.0		75.90 (17.63, 326.87)	
Model 4	REF = 1.0		106.08 (15.77, 713.75)	
**(b)**				
	**Q1 + Q2**	**Q3**	**Q4**	**Q5**
Median GRS6 (min, max)	−0.85 (−2.09, −0.21)	−0.13 (−0.21, 0.15)	0.48 (0.15, 0.88)	1.33 (0.89, 5.12)
XFGS/XFG cases (n = 118)	42	27	26	23
Person-years (%)	292,672 (40.0)	145,382 (20.0)	147,016 (20.0)	145,785 (20.0)
Model 1	0.82 (0.45, 1.52)	REF = 1.0	0.97 (0.45, 2.10)	0.71 (0.37, 1.38)
Model 2	0.94 (0.51, 1.72)	REF = 1.0	0.99 (0.47, 2.10)	0.82 (0.41, 1.63)
Model 3	0.94 (0.50, 1.79)	REF = 1.0	0.92 (0.39, 2.14)	0.79 (0.39, 1.62)
Model 4	0.93 (0.50, 1.72)	REF = 1.0	1.00 (0.49, 2.04)	0.83 (0.42, 1.65)
**(c)**				
	**Q1 + Q2**	**Q3**	**Q4**	**Q5**
Median GRS2 (min, max)	−0.88 (−2.96, −0.32)	0.36 (0.34, 0.39)	0.40 (0.40, 1.09)	1.12 (1.10, 1.15)
XFGS/XFG cases (n = 118)	6	24	25	63
Person-years (%)	292,121 (40.0)	154,440 (21.1)	139,422 (19.1)	144,872 (19.8)
Model 1	0.14 (0.04, 0.45)	REF = 1.0	2.06 (1.03, 4.11)	3.47 (1.82, 6.61)
Model 2	0.12 (0.04, 0.43)	REF = 1.0	2.24 (1.00, 4.98)	3.70 (1.93, 7.12)
Model 3	0.13 (0.05, 0.37)	REF = 1.0	2.69 (1.14, 6.34)	4.99 (2.62, 9.53)
Model 4	0.13 (0.04, 0.47)	REF = 1.0	2.44 (1.12, 5.31)	3.67 (1.90, 7.08)

Abbreviations: XFGS, exfoliation glaucoma suspect; XFG, exfoliation glaucoma; *LOXL1*, lysyl oxidase like 1; SNP, single nucleotide polymorphism; GRS6, genetic risk score constructed using 6 SNPs (rs12490863, rs10072088, rs4926244, rs7329408, rs11827818, rs3130283) excluding the two *LOXL1* SNPs rs1048661, rs3825942; GRS2, genetic risk score constructed using the two *LOXL1* SNPs rs1048661, rs3825942; REF, reference group; Q, quintile. Model 1: stratified by age in months, sex, and period at risk. Model 2: stratified by age in months, sex, period at risk, and adjusted for the following variables: ancestry (Scandinavian Caucasian, southern European Caucasian, other Caucasian, other races), self-reported history (yes/no) of hypercholesterolemia, hypertension, diabetes, and myocardial infarction; body mass index (kg/m^2^; <22, 22–23, 24–25, 26–27, 28–29, 30+), averaged intakes of total energy (kcal/day), caffeine (mg/day), folate intake (µg/day), and vitamin A (IU/day); pack-years of smoking (pack-years), alternate healthy eating index-2010, UV reflux (Robertson-Berger units, quintiles), latitude (°N, quintiles), physical activity (metabolic-equivalents of task-hour/week, quartiles). All adjusted covariates were included at baseline. Model 3: Model 2 + intraocular pressure > 25 mmHg. Model 4: Model 2 + family history of glaucoma.

**Table 5:** Harrell’s C-statistic (concordance) based on Cox regression models.

**Univariate Models**	**C-Index (95% CI)**
Model 1a: GRS8	0.76 (0.72, 0.79)
Model 1b: GRS2	0.73 (0.70, 0.77)
Model 1c: rs3825942	0.64 (0.62, 0.65)
Model 1d: rs1048661	0.63 (0.59, 0.68)
Model 1e: rs3825942 + rs1048661	0.76 (0.72, 0.79)
Model 1f: GRS6	0.51 (0.46, 0.57)
Model 1g: GRS6 + rs3825942 + rs1048661	0.76 (0.73, 0.80)
**Multivariable-Adjusted Models**	**C-Index (95% CI)**
Model 2: Age + sex + period at risk + age × sex	0.81 (0.77, 0.85)
Model 3: Age + sex + period at risk + age × sex + IOP > 25 mmHg	0.88 (0.84, 0.92)
Model 4: Age + sex + period at risk + age × sex + IOP > 25 mmHg + family history of glaucoma	0.88 (0.84, 0.92)
Model 5a: Model 4 + GRS8	0.93 (0.91, 0.95) ^1^
Model 5b: Model 4 + rs3825942	0.91 (0.89, 0.93)
Model 5c: Model 4 + rs1048661	0.90 (0.88, 0.92)
Model 5d: Model 4 + rs3825942 + rs1048661	0.93 (0.91, 0.95)
Model 5e: Model 4 + GRS2	0.93 (0.90, 0.95) ^2^
Model 5f: Model 4 + GRS6	0.88 (0.84, 0.92) ^3^
Model 5g: Model 4 + GRS6 + rs3825942 + rs1048661	0.93 (0.91, 0.95)

Abbreviations: GRS8, genetic risk score constructed using 8 single nucleotide polymorphisms (SNPs) (rs1048661, rs3825942, rs12490863, rs10072088, rs4926244, rs7329408, rs11827818, rs3130283); GRS2, genetic risk score constructed using the two *LOXL1* SNPs rs1048661, rs3825942; GRS6, genetic risk score constructed using 6 SNPs (rs12490863, rs10072088, rs4926244, rs7329408, rs11827818, rs3130283), excluding the two *LOXL1* SNPs rs1048661, rs3825942; IOP, intraocular pressure. ^1^ Comparing Model 5a to Model 4, the C-index increase was statistically significant (*p* = 0.04 based on the DeLong test). ^2^ Comparing Model 5e to Model 4, the C-index increase was statistically significant (*p* = 0.03 based on the DeLong test). ^3^ Comparing Model 5f to Model 4, the C-index increase was not significant (*p* = 0.99 based on the DeLong test).

## Data Availability

The data presented in this study are available on request from the corresponding author and completion of a request form that can be found in https://www.nurseshealthstudy.org/researchers (accessed on 17 August 2025), due to participant confidentiality and privacy concerns.

## Supplementary Materials

The following supporting information can be downloaded at: https://www.mdpi.com/article/10.3390/metabo15090582/s1, Supplementary Methods; Table S1: Age and age-adjusted characteristics of participants with metabolomics data available (n = 7547) by GRS tertiles from NHS (1989–1990), NHS2 (1996–1999), and HPFS (1993–1995) as of blood draw; Table S2a: Harrell’s C-statistic (concordance) based on Cox regression models for XFG cases only (XFG cases: n = 58); Table S2b: Harrell’s C-statistic (concordance) based on Cox regression models for XFGS cases only (XFGS cases: n = 60); Figure S1: Individual metabolites among 427 metabolites evaluated that were NEF < 0.2 for the adjusted linear regression model (n = 7547) for GRS8, GRS6, GRS2, and the 8 component SNPs; Figure S2: Comparison of the associations of the individual metabolites included in the bile acids metabolite class with GRS8, GRS6, GRS2, and rs3825942; Figure S3: Comparison of the associations of the individual metabolites included in the fatty acyls metabolite class with GRS8, GRS6, GRS2, and rs3825942; Figure S4: Comparison of the associations of the individual metabolites included in the triglycerides with ≥3 DB metabolite class with GRS8, GRS6, and rs1048661; Figure S5: Metabolite classes (n = 20) evaluated in the adjusted linear regression models (n = 7547) for GRS8, GRS6, GRS2, and component SNPs; Figure S6: Secondary analysis by age (≤56 (n = 3740) vs. >56 years (n = 3807)) for individual metabolites that were NEF < 0.2 for the adjusted linear regression model for GRS8, GRS6, GRS2, and *LOXL1* SNPs; Figure S7: Secondary analysis by sex (female (n = 5894) vs. male (n = 1653)) for individual metabolites that were NEF < 0.2 for the adjusted linear regression model for GRS8, GRS6, GRS2, and *LOXL1* SNPs; Figure S8: Secondary analysis by latitude (<41° N (n = 3967) vs. ≥41° N (n = 3580)) for individual metabolites that were NEF < 0.2 for the adjusted linear regression model for GRS8, GRS6, GRS2, and *LOXL1* SNPs.
