# Supplementary material for: Metabolomic Signatures and Predictive Utility of LOXL1-Associated Genetic Risk Scores for Exfoliation Syndrome/Glaucoma in US Cohorts
Source: Metabolites. 2025 Aug 30;15(9):582. doi: 10.3390/metabo15090582 (PMC12472000; doi:10.3390/metabo15090582)
Supplement: Supplementary file 1 [file metabolites-15-00582-s001.zip › Supplementary materials.pdf]

SUPPLEMENTARY MATERIALS

Supplementary methods

Table S1. Age and age-adjusted characteristics of participants with metabolomics data available (n=7547) by GRS tertiles from NHS (1989-1990), NHS2 (1996-1999), and HPFS (1993-1995) as of blood draw

Table S2 a. Harrell's C-statistic (concordance) based on Cox regression models for XFG cases only (XFG cases: n=58)

Table S2 b. Harrell's C-statistic (concordance) based on Cox regression models for XFGS cases only (XFGS cases: n=60)

Figure S1. Individual metabolites among 427 metabolites evaluated that were NEF<0.2 for the adjusted linear regression model (n=7547) for GRS8, GRS6, GRS2, and the 8 component SNPs.

Figure S2. Comparison of the associations of the individual metabolites included in the bile acids metabolite class with GRS8, GRS6, GRS2, and rs3825942.

Figure S3. Comparison of the associations of the individual metabolites included in the fatty acyls metabolite class with GRS8, GRS6, GRS2, and rs3825942

Figure S4. Comparison of the associations of the individual metabolites included in the triglycerides with ≥3 DB metabolite class with GRS8, GRS6, and rs1048661.

Figure S5. Metabolite classes (n=20) evaluated in the adjusted linear regression models (n=7547) for GRS8, GRS6, GRS2, and component SNPs.

Figure S6. Secondary analysis by age (≤ 56 (n=3740) vs. > 56 years (n=3807)) for individual metabolites that were NEF<0.2 for the adjusted linear regression model for GRS8, GRS6, GRS2, and *LOXL1* SNPs.

Figure S7. Secondary analysis by sex (female (n=5894) vs. male (n=1653)) for individual metabolites that were NEF<0.2 for the adjusted linear regression model for GRS8, GRS6, GRS2, and *LOXL1* SNPs.

Figure S8. Secondary analysis by latitude (<41°N (n=3967) vs. ≥41°N (n=3580)) for individual metabolites that were NEF<0.2 for the adjusted linear regression model for GRS8, GRS6, GRS2, and *LOXL1* SNPs.

Supplementary methods

Genotyping and GRS calculation

Genomic datasets, genotype imputation, and quality control procedures were conducted separately for each cohort with details described in earlier studies[1, 2]. In summary, participants came from five subpopulations, each genotyped at different times using distinct platforms. Specifically: 1) Affymetrix group: Genome-wide Human SNP Array 6.0; 2) Illumina group: HumanHap300 BeadChip, HumanHap550-Quad BeadChip, Human610-Quad BeadChip, or Human660W-Quad BeadChip; 3) OmniExpress group: Illumina HumanOmniExpress-12 BeadChip; 4) OncoArray group: Infinium OncoArray-550K BeadChip; 5) HumanCore group: Illumina HumanCoreExome-12v1-0 BeadChip. Variants with a call rate below 95% were excluded. Duplicate samples were identified through pairwise identity-by-descent (IBD) analysis. When individuals were genotyped more than once on different platforms, one duplicate was retained following the priority order: Affymetrix, Illumina, OmniExpress, OncoArray, HumanCore. If two individuals from different cohorts showed high genetic concordance, both were excluded. Genotype phasing was carried out using ShapeIT (v2.r837), and imputation was performed with Minimac3 using the 1000 Genomes Project Phase 3 Integrated Release Version 5 as the reference panel, yielding approximately 47 million variants[3, 4].

The genetic risk score (GRS) for exfoliation glaucoma (XFG) was derived from the largest genome-wide association study (GWAS) on exfoliation syndrome (XFS), which included 9,035 cases and 17,008 controls in the discovery phase, and 4,803 cases with 93,267 controls in the replication phase. This GWAS identified eight variants across seven genes (LOXL1, CACNA1A, POMP, TMEM136, AGPAT1, SEMA6A, and RBMS3) that reached genome-wide significance ($P < 5\times10^{-8}$)[5].

Within the NHS/NHS2/HPFS datasets, each of these eight variants was coded by the imputed fractional dosage of the risk allele, with the imputation values ranging from zero for no risk allele to 2 for two copies. For rs10072088, the dosage of the T allele was used, and its beta coefficient from the GWAS was multiplied by −1 to reflect the correct direction of association. The XFS GRS was then calculated as the weighted sum of the risk allele dosages multiplied by the effect sizes reported in the GWAS for the eight SNPs. The following table presents the information on the SNPs included in the GRS calculation.

SNPs included in the GRS calculation

RSID	Chr	position	Gene	Effect/reference allele	Odds ratio[5]	Beta[5]
rs1048661	15	73927205	*LOXL1*	G/T	1.99	0.69
rs3825942	15	74219582	*LOXL1*	G/A	6.48	1.87
rs12490863	3	29907310	*RBMS3*	A/G	1.19	0.17
rs10072088	5	116019417	*SEMA6A*	C/T	0.90	-0.11
rs4926244	19	13374913	*CACNA1A*	C/T	1.13	0.12
rs7329408	13	29166671	*FLT1-POMP*	T/C	1.22	0.20
rs11827818	11	120198728	*TMEM136*	G/A	1.14	0.13
rs3130283	6	32138545	*AGPAT1*	A/C	1.13	0.12

Abbreviations: Chr, chromosome; RSID, rs identification.

Metabolomic profiling

Water-soluble metabolites were analyzed in positive ionization mode using hydrophilic interaction liquid chromatography (HILIC) coupled to mass spectrometry. This setup consisted of a Shimadzu Nexera X2 U-HPLC (Shimadzu Corp., Marlborough, MA) connected to a Q Exactive mass spectrometer (Thermo Fisher Scientific, Waltham, MA), hereafter referred to as HILIC-positive. Plasma samples (10 µL) were extracted with 90 µL of acetonitrile/methanol/formic acid (74.9:24.9:0.2, v/v/v) containing stable isotope-labeled internal standards (valine-d8, Sigma-Aldrich, St. Louis, MO; and phenylalanine-d8, Cambridge Isotope Laboratories, Andover, MA). Extracts were centrifuged (10 min, 9,000 × g, 4 °C), and the supernatant was injected onto a 150 × 2 mm, 3 µm Atlantis HILIC column (Waters, Milford, MA). The column was initially eluted isocratically with 5% mobile phase A (10 mM ammonium formate and 0.1% formic acid in water) for 0.5 min, followed by a 10-minute linear gradient to 40% mobile phase B (acetonitrile with 0.1% formic acid) at a flow rate of 250 µL/min.

Mass spectrometry was performed using electrospray ionization in positive mode, acquiring full-scan data over 70–800 m/z at 70,000 resolution and 3 Hz. Instrument settings included sheath gas at 40, sweep gas at 2, spray voltage at 3.5 kV, capillary temperature at 350 °C, S-lens RF at 40, heater temperature at 300 °C, 1 microscan, AGC target of 1×10^6, and a maximum ion time of 250 ms.

For lipid profiling (C8-positive method), plasma (10 µL) was extracted with 190 µL of isopropanol containing 1,2-didodecanoyl-sn-glycero-3-phosphocholine (Avanti Polar Lipids, Alabaster, AL). Following centrifugation, supernatants were injected onto a 100 × 2.1 mm, 1.7 µm ACQUITY BEH C8 column (Waters). Chromatographic separation began with 80% mobile phase A (95:5:0.1, v/v/v 10 mM ammonium acetate/methanol/formic acid) for 1 min, followed by a linear gradient to 80% mobile phase B (99.9:0.1, v/v methanol/formic acid) over 2 min, then to 100% B over the next 7 min, holding at 100% B for 3 min.

Lipid MS analysis used electrospray ionization in positive mode with full-scan acquisition from 200–1100 m/z at 70,000 resolution and 3 Hz. Settings included sheath gas at 50, in-source CID at 5 eV, sweep gas at 5, spray voltage at 3 kV, capillary temperature at 300 °C, S-lens RF at 60, heater temperature at 300 °C, 1 microscan, AGC target of 1×10^6, and maximum ion time of 100 ms. Lipid species were annotated by total acyl carbon number and total double bond number.

Raw Orbitrap MS data were processed using TraceFinder 3.3 (Thermo Fisher Scientific) and Progenesis QI v1.0.5165.27075 (Nonlinear Dynamics, Newcastle upon Tyne, UK). Quality control included: (1) analysis of pooled plasma reference samples and synthetic metabolite mixtures at the start and end of each run to monitor instrument stability, (2) internal standard evaluation for injection consistency, and (3) inclusion of pooled plasma QC samples every ~5% of the run to assess metabolite repeatability.

Plasma samples were thawed on ice prior to aliquoting. During aliquot preparation, an additional 10 µL from each sample was combined into a pooled plasma QC sample, maintained on dry ice to ensure rapid freezing, and stored at −80 °C between batches. Once complete, pooled plasma was thawed on ice, vortexed, and divided into sub-aliquots for each LC-MS method.

Metabolite identities were confirmed using authentic reference standards that matched retention time, m/z, and MS/MS spectra from prior human plasma identifications, as well as pooled reference samples. LC methods employed different stationary phase chemistries to reproducibly separate metabolites based on physical properties, while MS provided m/z resolution and quantification across a wide dynamic range. Method development was guided by reference standards to define chromatographic retention times, multiple reaction monitoring transitions, de-clustering potentials, and collision energies. Data analysis combined automated software outputs with manual peak verification. Dr. Clary Clish, who leads the MIT laboratory where these analyses were conducted, has over a decade of experience in metabolite species identification.

Inverse probability weighting

To account for potential selection bias arising from the process of inclusion in the GWAS studies that generated the genetic data, we applied inverse probability weighting (IPW) across the three cohorts. The probability of selection (propensity score) was estimated for each participant using logistic regression, with selection status as the dependent variable and relevant demographic and clinical variables as predictors (including age at blood collection, cohort, follow-up interval, and status of various medical conditions: breast cancer, myocardial infarction, colon cancer, glaucoma, gout, skin cancer, ovarian cancer, pancreatic cancer, diabetes, pulmonary embolism, kidney stone, uterine or endometrial cancer, benign breast disease, endometriosis, post-traumatic stress disorder, and prostate cancer). Predicted probabilities of selection were obtained from this model for each participant. IPW weights were then calculated as the inverse of the estimated probability of selection. These weights were applied in subsequent analyses to produce estimates that are less biased by differential selection into the genetic data subset.

Table S1. Age and age-adjusted characteristics of participants with metabolomics data available (n=7547) by GRS8 tertiles from NHS (1989-1990), NHS2 (1996-1999), and HPFS (1993-1995) as of blood draw

**Characteristics**[a]	**Tertile 1 (n=2516)**	**Tertile 2 (n=2518)**	**Tertile 3 (n=2513)**
Genetic risk score	-1.173 (0.619)	0.224 (0.233)	1.011 (0.237)
Age, years[b]	56.0 (9.3)	56.2 (9.1)	56.1 (9.2)
Female, %	78.2	79.4	78.3
Scandinavian ancestry, %	8.5	9.5	8.5
Family history of glaucoma, %[b]	14.7	15.8	16.3
Self-reported diabetes diagnosis, %	3.6	3.4	4.3
Self-reported hypertension diagnosis, %	24.6	24.2	26.0
Self-reported high cholesterol diagnosis, %	30.1	30.2	32.3
Self-reported history of myocardial infarction, %	2.5	2.0	2.4
Total calories, kcal/day	1795 (477)	1784 (482)	1801 (482)
Total vitamin A intake, IU/day	13502 (6761)	13696 (7180)	13665 (6979)
Total caffeine intake, mg/day	285 (212)	286 (208)	285 (206)
Folate intake, µg/day	431 (200)	421 (201)	424 (192)
Weighted lifetime average latitude of residence, ºN	39.5 (3.8)	39.4 (3.8)	39.4 (3.9)
Annual UV flux, $\times 10^{-4}$ mW/m^2	186.5 (26.5)	187.8 (27.1)	186.9 (26.4)
Total alcoholic intake, g/day	7.3 (11.2)	7.1 (10.3)	7.0 (10.4)
Cigarette smoking, pack-years	10.1 (16.0)	10.1 (15.9)	10.3 (16.4)
Body mass index, kg/m^2	24.9 (4.0)	24.8 (4.0)	25.1 (4.4)
In top 25th percentile for physical activity, %	24.3	24.5	24.6
Alternate healthy eating index 2010	45.9 (9.6)	46.3 (9.5)	46.0 (9.7)

Abbreviations: SD, standard deviation; UV, ultraviolet

[a] Values are presented as means (SD) for continuous variables and percentages for categorical variables.

[b] All values other than age have been directly standardized to the age distribution of all the participants.

Table S2 a. Harrell's C-statistic (concordance) based on Cox regression models for XFG cases only (XFG cases: n=58)

Univariate Models	C-index (95 % CI)
Model 1a: GRS8	0.73 (0.68, 0.79)
Model 1b: GRS2	0.72 (0.66, 0.77)
Model 1c: rs3825942	0.63 (0.60, 0.66)
Model 1d: rs1048661	0.62 (0.55, 0.68)
Model 1e: rs3825942 + rs1048661	0.73 (0.68, 0.79)
Model 1f: GRS6	0.50 (0.42, 0.58)
Model 1g: GRS6 + rs3825942 + rs1048661	0.74 (0.68, 0.79)
**Multivariable-adjusted Models**	**C-index (95 % CI)**
Model 2: Age + sex + period at risk + age*sex	0.83 (0.78, 0.87)
Model 3: Age + sex + period at risk + age*sex + IOP>25 mmHg	0.91 (0.87, 0.94)
Model 4: Age + sex + period at risk + age*sex + IOP>25 mmHg + family history of glaucoma	0.90 (0.87, 0.94)
Model 5a: Model 4 + GRS8	0.93 (0.89, 0.96)
Model 5b: Model 4 + rs3825942	0.91 (0.87, 0.95)
Model 5c: Model 4 + rs1048661	0.92 (0.89, 0.95)
Model 5d: Model 4 + rs3825942 + rs1048661	0.93 (0.90, 0.96)
Model 5e: Model 4 + GRS2	0.93 (0.89, 0.96)
Model 5f: Model 4 + GRS6	0.90 (0.87, 0.94)
Model 5g: Model 4 + GRS6 + rs3825942 + rs1048661	0.93 (0.90, 0.96)

Abbreviations: SE, standard error; IOP, intraocular pressure; GRS8, genetic risk score constructed using 8 SNPS (rs1048661, rs3825942, rs12490863, rs10072088, rs4926244, rs7329408, rs11827818, rs3130283); GRS6, genetic risk score constructed using 6 SNPS (rs12490863, rs10072088, rs4926244, rs7329408, rs11827818, rs3130283), excluding the two *LOXL1* SNPs rs1048661, rs3825942.

Table S2 b. Harrell's C-statistic (concordance) based on Cox regression models for XFGS cases only (XFGS cases: n=60)

Univariate Models	C-index (95 % CI)
Model 1a: GRS8	0.78 (0.74, 0.83)
Model 1b: GRS2	0.75 (0.71, 0.79)
Model 1c: rs3825942	0.65 (0.65, 0.65)
Model 1d: rs1048661	0.65 (0.59, 0.71)
Model 1e: rs3825942 + rs1048661	0.78 (0.74, 0.82)
Model 1f: GRS6	0.53 (0.44, 0.61)
Model 1g: GRS6 + rs3825942 + rs1048661	0.79 (0.75, 0.83)
**Multivariable-adjusted Models**	**C-index (95 % CI)**
Model 2: Age + sex + period at risk + age*sex	0.80 (0.75, 0.85)
Model 3: Age + sex + period at risk + age*sex + IOP>25 mmHg	0.87 (0.82, 0.92)
Model 4: Age + sex + period at risk + age*sex + IOP>25 mmHg + family history of glaucoma	0.87 (0.82, 0.92)
Model 5a: Model 4 + GRS8	0.93 (0.89, 0.96)
Model 5b: Model 4 + rs3825942	0.91 (0.87, 0.94)
Model 5c: Model 4 + rs1048661	0.89 (0.85, 0.93)
Model 5d: Model 4 + rs3825942 + rs1048661	0.93 (0.90, 0.96)
Model 5e: Model 4 + GRS2	0.93 (0.90, 0.96)
Model 5f: Model 4 + GRS6	0.87 (0.82, 0.92)
Model 5g: Model 4 + GRS6 + rs3825942 + rs1048661	0.93 (0.90, 0.96)

Abbreviations: SE, standard error; IOP, intraocular pressure; GRS8, genetic risk score constructed using 8 SNPS (rs1048661, rs3825942, rs12490863, rs10072088, rs4926244, rs7329408, rs11827818, rs3130283); GRS6, genetic risk score constructed using 6 SNPS (rs12490863, rs10072088, rs4926244, rs7329408, rs11827818, rs3130283), excluding the two *LOXL1* SNPs rs1048661, rs3825942.

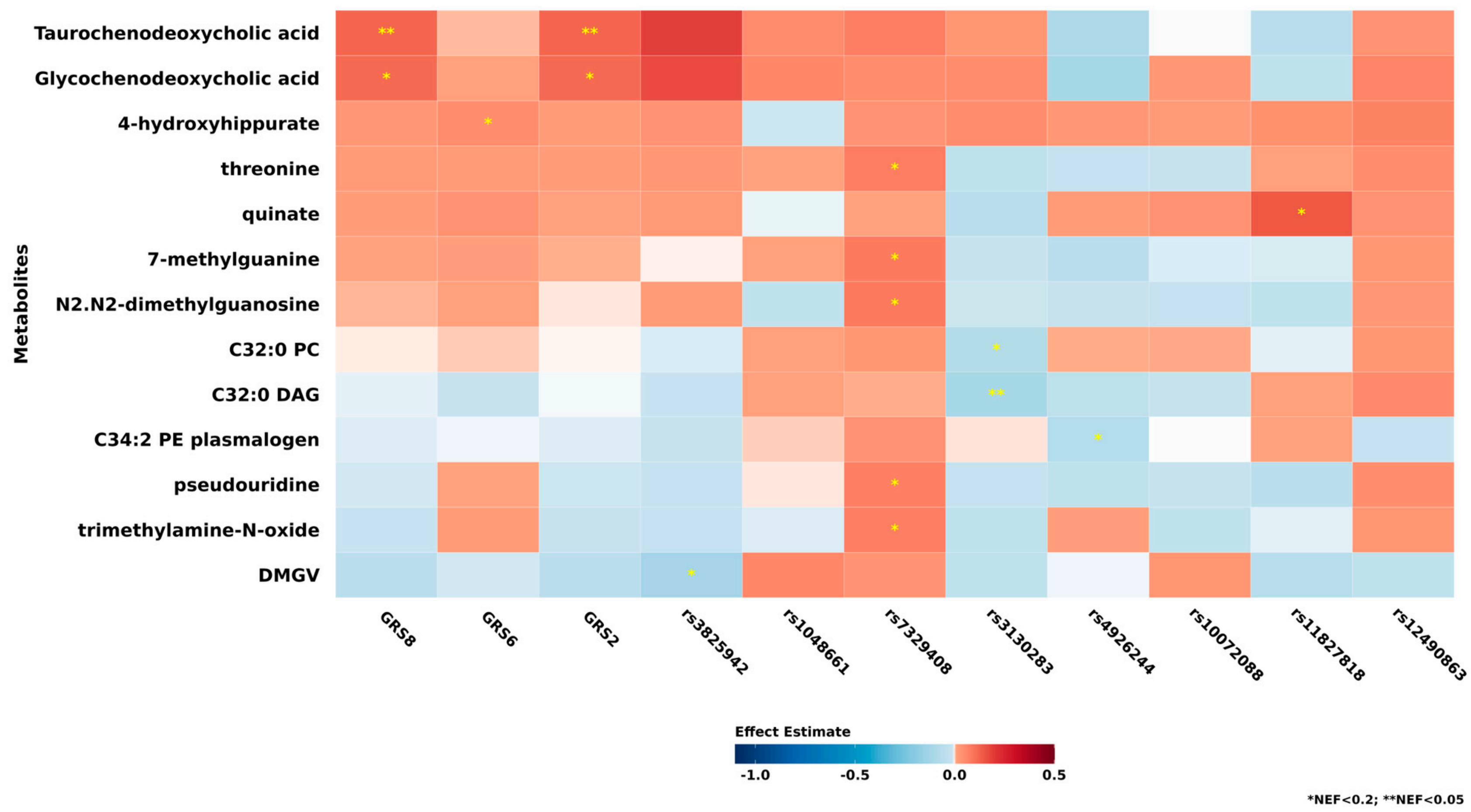

Figure S1. Individual metabolites among 427 metabolites evaluated that were NEF<0.2 for the adjusted linear regression model (n=7547) for GRS8, GRS6, GRS2, and the 8 component SNPs.

All models adjusted for age, fasting status, family history of glaucoma (yes/no), self-reported history (yes/no) of hypercholesterolemia, hypertension, diabetes, and myocardial infarction, body mass index (kg/m^2), total energy intake (kcal/day), caffeine intake (mg/day), folate intake (µg/day), alcohol intake (g/day), vitamin A intake (IU/day), pack-years of smoking (pack-years), Alternate Health Eating Index (0-100 score), UV flux (mW/m^2), latitude (°N), physical activity (metabolic-equivalents of task-hour/week, quintiles), principal components 1 to 10.

* NEF < 0.2; ** NEF < 0.05.

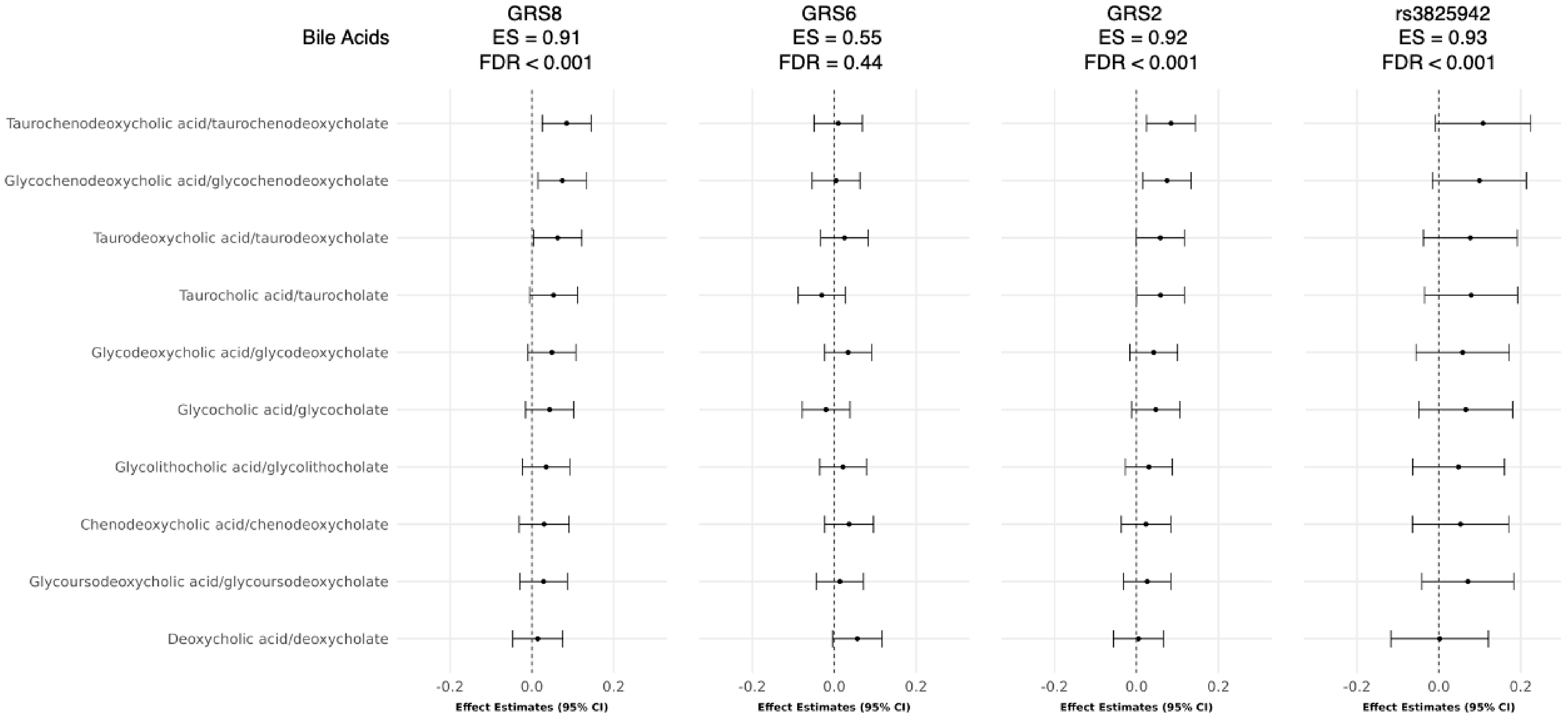

Figure S2. Comparison of the associations of the individual metabolites included in the bile acids metabolite class with GRS8, GRS6, GRS2, and rs3825942. Models adjusted for age, fasting status, and top 10 principal components. ES, enrichment score; FDR, false discovery rate.

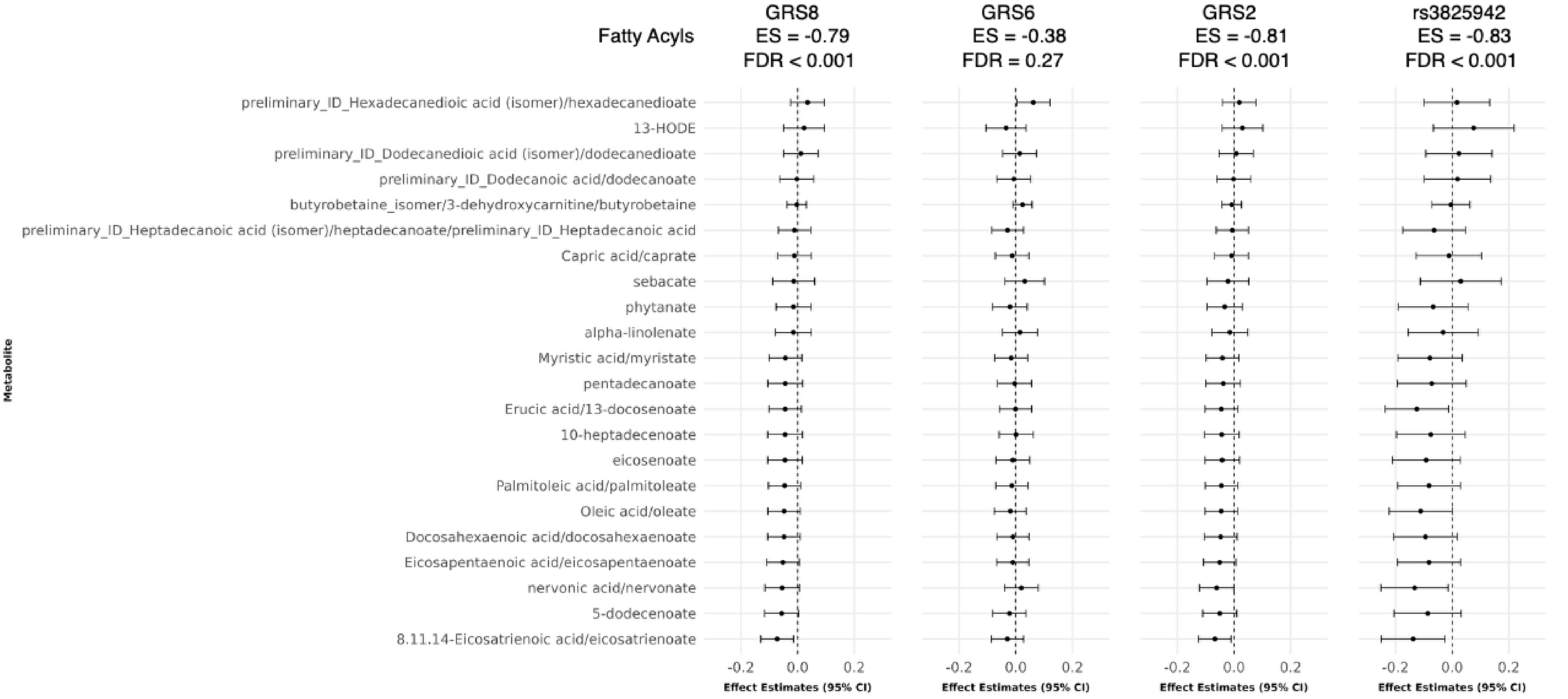

Figure S3. Comparison of the associations of the individual metabolites included in the fatty acyls metabolite class with GRS8, GRS6, GRS2, and rs3825942. Models adjusted for age, fasting status, and top 10 principal components. ES, enrichment score; FDR, false discovery rate.

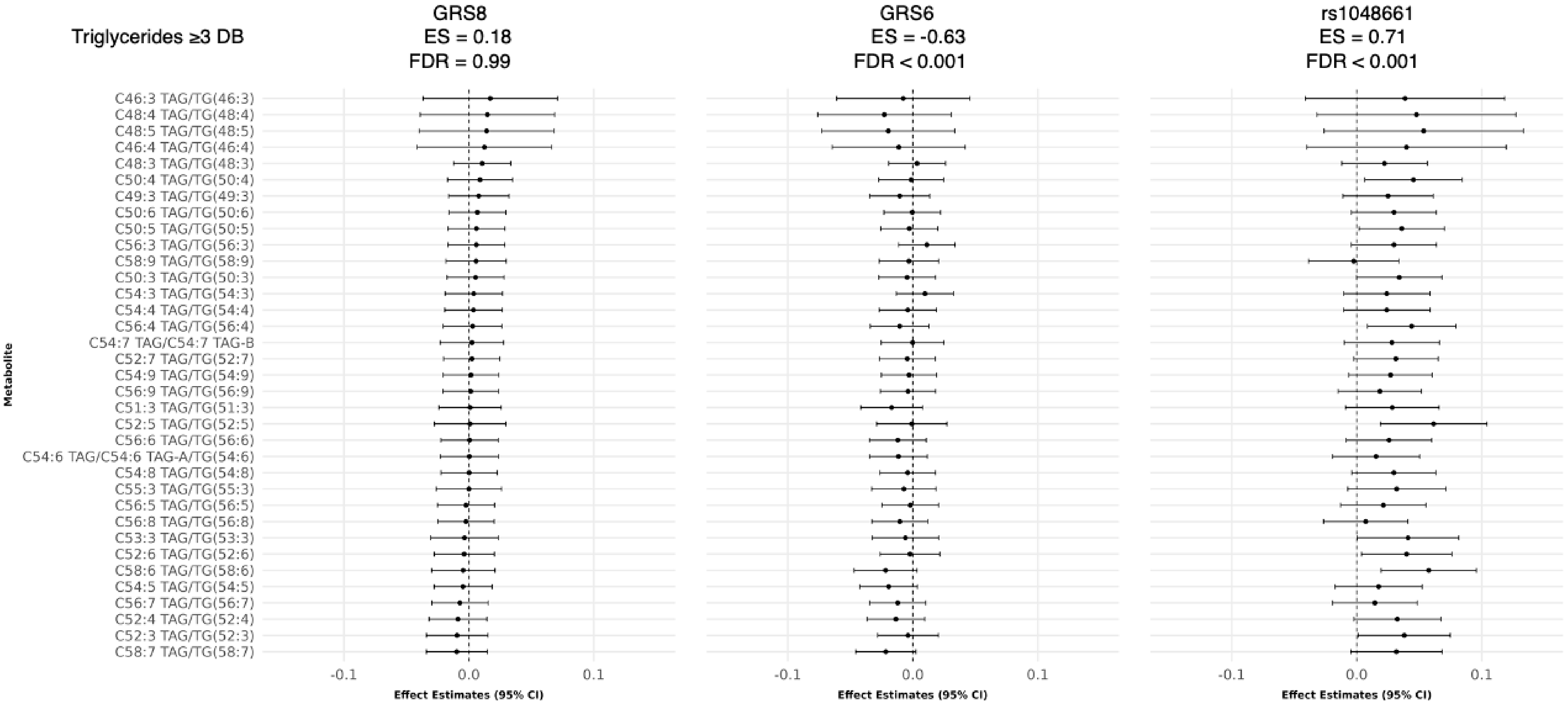

Figure S4. Comparison of the associations of the individual metabolites included in the triglycerides with ≥3 DB metabolite class with GRS8, GRS6, and rs1048661. Models adjusted for age, fasting status, and top 10 principal components. ES, enrichment score; FDR, false discovery rate.

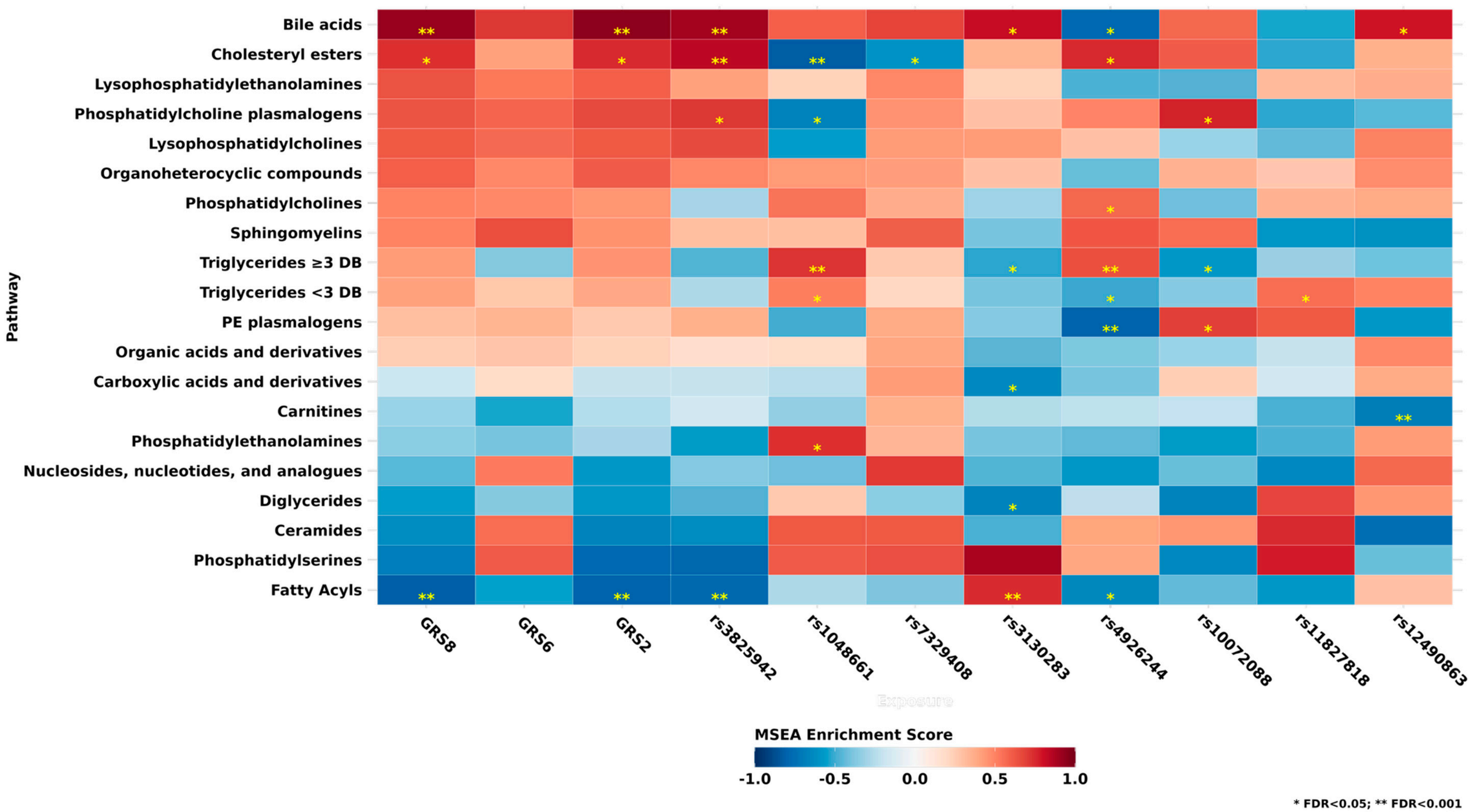

Figure S5. Metabolite classes (n=20) evaluated in the adjusted linear regression models (n=7547) for GRS8, GRS6, GRS2, and component SNPs.

All models adjusted for age, fasting status, family history of glaucoma (yes/no), self-reported history (yes/no) of hypercholesterolemia, hypertension, diabetes, and myocardial infarction, body mass index (kg/m^2), total energy intake (kcal/day), caffeine intake (mg/day), folate intake (µg/day), alcohol intake (g/day), vitamin A intake (IU/day), pack-years of smoking (pack-years), Alternate Health Eating Index (0-100 score), UV flux (mW/m^2), latitude (ºN), physical activity (metabolic-equivalents of task-hour/week, quintiles), principal components 1 to 10.

* FDR < 0.05; ** FDR < 0.001.

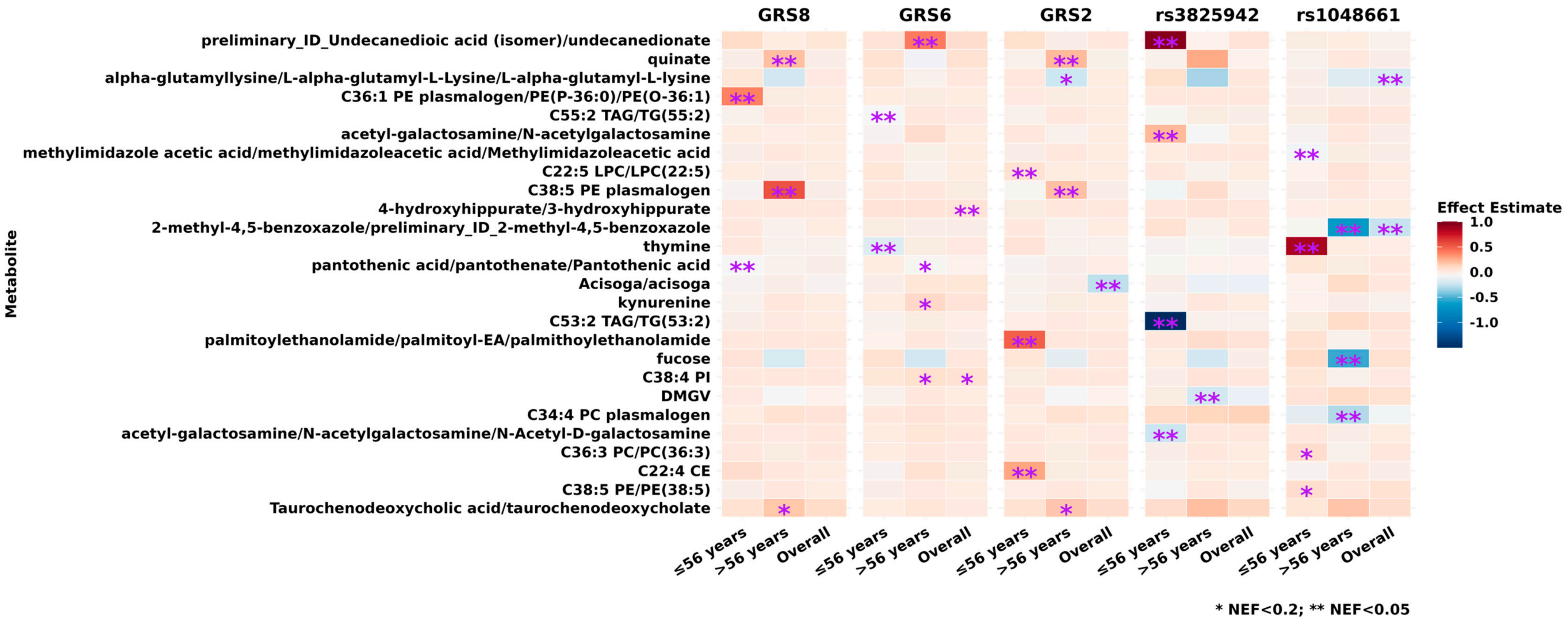

Figure S6. Secondary analysis by age (≤ 56 (n=3740) vs. > 56 years (n=3807)) for individual metabolites that were NEF<0.2 for the adjusted linear regression model for GRS8, GRS6, GRS2, and *LOXL1* SNPs. All models adjusted for age, fasting status, and top 10 principal components.

* NEF < 0.2; ** NEF < 0.05.

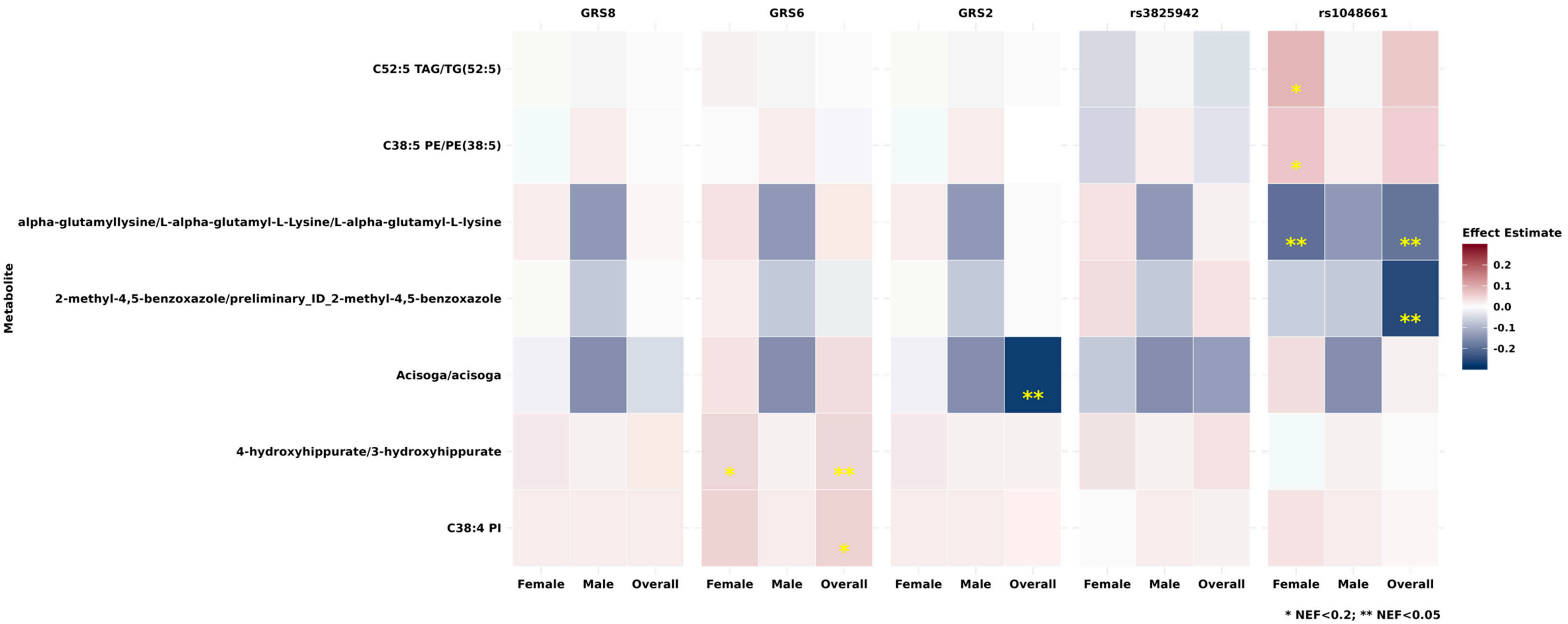

Figure S7. Secondary analysis by sex (female (n=5894) vs. male (n=1653)) for individual metabolites that were NEF<0.2 for the adjusted linear regression model for GRS8, GRS6, GRS2, and *LOXL1* SNPs. All models adjusted for age, fasting status, and top 10 principal components.

* NEF < 0.2; ** NEF < 0.05.

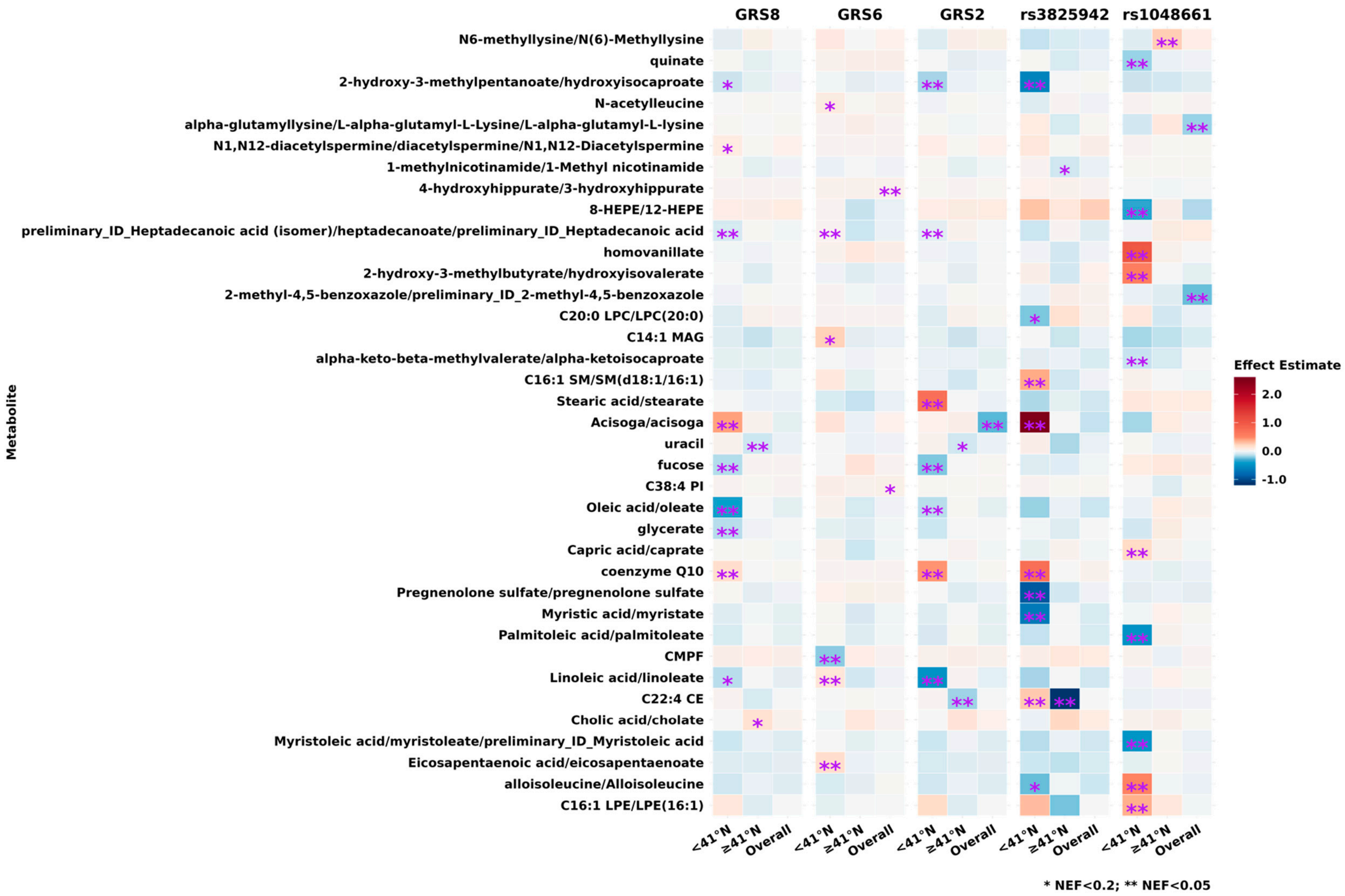

Figure S8. Secondary analysis by latitude (<41°N (n=3967) vs. ≥41°N (n=3580)) for individual metabolites that were NEF<0.2 for the adjusted linear regression model for GRS8, GRS6, GRS2, and *LOXL1* SNPs. All models adjusted for age, fasting status, and top 10 principal components.

* NEF < 0.2; ** NEF < 0.05.
